# Pandemic Influenza A Viruses Escape from Restriction by Human MxA through Adaptive Mutations in the Nucleoprotein

**DOI:** 10.1371/journal.ppat.1003279

**Published:** 2013-03-28

**Authors:** Benjamin Mänz, Dominik Dornfeld, Veronika Götz, Roland Zell, Petra Zimmermann, Otto Haller, Georg Kochs, Martin Schwemmle

**Affiliations:** 1 Department of Virology, Institute for Medical Microbiology and Hygiene, University of Freiburg, Freiburg, Germany; 2 Institute of Virology und Antiviral Therapy, Universitätsklinikum Jena, Friedrich Schiller University Jena, Jena, Germany; Centro Nacional de Biotecnologia (CSIC) and CIBER de Enfermedades Respiratorias, Spain

## Abstract

The interferon-induced dynamin-like MxA GTPase restricts the replication of influenza A viruses. We identified adaptive mutations in the nucleoprotein (NP) of pandemic strains A/Brevig Mission/1/1918 (1918) and A/Hamburg/4/2009 (pH1N1) that confer MxA resistance. These resistance-associated amino acids in NP differ between the two strains but form a similar discrete surface-exposed cluster in the body domain of NP, indicating that MxA resistance evolved independently. The 1918 cluster was conserved in all descendent strains of seasonal influenza viruses. Introduction of this cluster into the NP of the MxA-sensitive influenza virus A/Thailand/1(KAN-1)/04 (H5N1) resulted in a gain of MxA resistance coupled with a decrease in viral replication fitness. Conversely, introduction of MxA-sensitive amino acids into pH1N1 NP enhanced viral growth in Mx-negative cells. We conclude that human MxA represents a barrier against zoonotic introduction of avian influenza viruses and that adaptive mutations in the viral NP should be carefully monitored.

## Author Summary

Influenza A viruses of avian or swine origin sporadically enter into the human population but do not transmit between individuals. In rare cases, however, they establish a new virus lineage in humans. The mechanisms by which invading viruses overcome the species barrier are not well understood, but multiple adaptations to the new host are required. Surprisingly little is known about adaptive mutations that overcome restriction factors of the intrinsic and innate host defense system. In this study, we have identified adaptive mutations in pandemic strains A/Brevig Mission/1/1918 and A/Hamburg/4/2009 that confer resistance to the interferon-induced antiviral factor MxA which is a dynamin-like large GTPase that recognizes the incoming viral nucleocapsids and blocks their function. The resistance-enhancing mutations changed several amino acids in the viral nucleoprotein which is the main nucleocapsid component. These mutations were sufficient to increase the pathogenicity of an avian influenza virus strain in a Mx-positive mouse model. Interestingly, the resistance-associated amino acids are counter-selected in circulating avian influenza strains, because they compromise general viral replication fitness. The present data indicate that the innate immunity factor MxA provides a barrier against zoonotic introduction of influenza A viruses and that adaptive mutations in the nucleoprotein must be carefully monitored.

## Introduction

Avian influenza A viruses sporadically transmit from waterfowl, their natural reservoir, into the human population [1]–[5]. These zoonotic viruses usually cannot propagate in the new human host, nor do they readily transmit between humans [6]–[8]. In rare cases, however, influenza A viruses of avian origin break the species barrier and establish new virus lineages in humans. In the last 100 years, the introduction of an influenza A virus with a novel nucleoprotein (NP) gene segment occurred only on two occasions, both of which led to pandemics: in 1918 (“Spanish” H1N1) an avian virus and in 2009 (pH1N1) a reassortant virus (comprising gene segments of two swine influenza viruses) established a stable lineage in humans [9], [10]. In contrast, the 1957 (“Asian” H2N2) and the 1968 (“Hong-Kong” H3N2) pandemics were caused by genetic reassortment events, whereby the circulating human strains acquired some gene segments from avian sources but kept, among others, their 1918-derived NP [11].

To overcome the species barrier, multiple adaptations to the new host are required [6]. Theoretically, two categories of adaptive mechanisms can be envisaged. One comprises adaptations to cellular factors which promote viral infection yet differ between hosts. These include, for example, changes in the viral hemagglutinin during the adaptation of avian influenza A viruses to humans [12], or altered binding of viral proteins to different cellular importins [11], [13]. The second category comprises adaptations to counteract cellular restriction factors that inhibit virus replication. These factors are part of the intrinsic and innate host defense mechanisms and may exert a strong selective pressure against newly invading viruses. Surprisingly little is known about adaptive mutations that overcome such host restriction factors and facilitate trans-species transmission of influenza viruses.

The human interferon (IFN) system represents a major innate defense against zoonotic viruses. Among the many antiviral factors induced by IFNs, the MxA protein is one of the most potent characterized to date [14]. It is a key effector molecule inhibiting influenza A virus as well as several other human RNA viruses [15], [16]. MxA is a dynamin-like large GTPase which consists of an N-terminal globular GTPase domain, a bundle signaling element, and a C-terminal helical stalk. The recent atomic resolution of the MxA structure revealed that it forms stable tetramers and oligomers which assemble in a criss-cross manner via the stalk [17], [18]. A current model proposes that, upon viral infection, MxA recognizes the incoming vRNPs and starts to self-assemble into rings, resulting in a higher-order oligomeric complex that blocks vRNP function [18], [19].

In accordance with this model, recent findings suggest that NP determines the relative sensitivity of influenza A viruses toward the antiviral action of MxA. Avian influenza viruses were found to be generally more sensitive to MxA than human strains [20], which was subsequently shown through reassortant viruses to be dependent on the origin of NP [21]. These findings suggest that human strains acquire adaptive mutations in NP to evade MxA restriction.

Here, we identified the amino acids critical for MxA resistance in the two NP proteins introduced into the human population in 1918 (by the “Spanish” H1N1 influenza A virus) and in 2009 (by the pH1N1 strain). These residues clustered into two distinct but overlapping “patches” in the body domain of the protein. Introduction of these amino acids into an MxA-sensitive H5N1 NP was sufficient to render the avian polymerase resistant to MxA. Surprisingly, the resistance-associated substitutions resulted in impaired viral growth in both mammalian and avian cells when introduced into recombinant H5N1 virus A/Thailand/1(KAN-1)/04. The amino acid clusters identified here are highly conserved in circulating human isolates and virtually absent in NPs of avian influenza A viruses. Several of the amino acids that confer increased resistance to human MxA are also conserved in influenza A viruses of the classical swine lineage, correlating with resistance of these viruses to swine Mx1. These findings suggest that multiple adaptive amino acid changes would be required for H5N1 viruses to both escape from MxA restriction and maintain viral fitness. Partial adaptation in an intermediate host, such as the pig, might facilitate this demanding process.

## Results

### Identification of residues in NP of the pandemic 1918 influenza A virus responsible for resistance to murine Mx1

**Figure 1:**
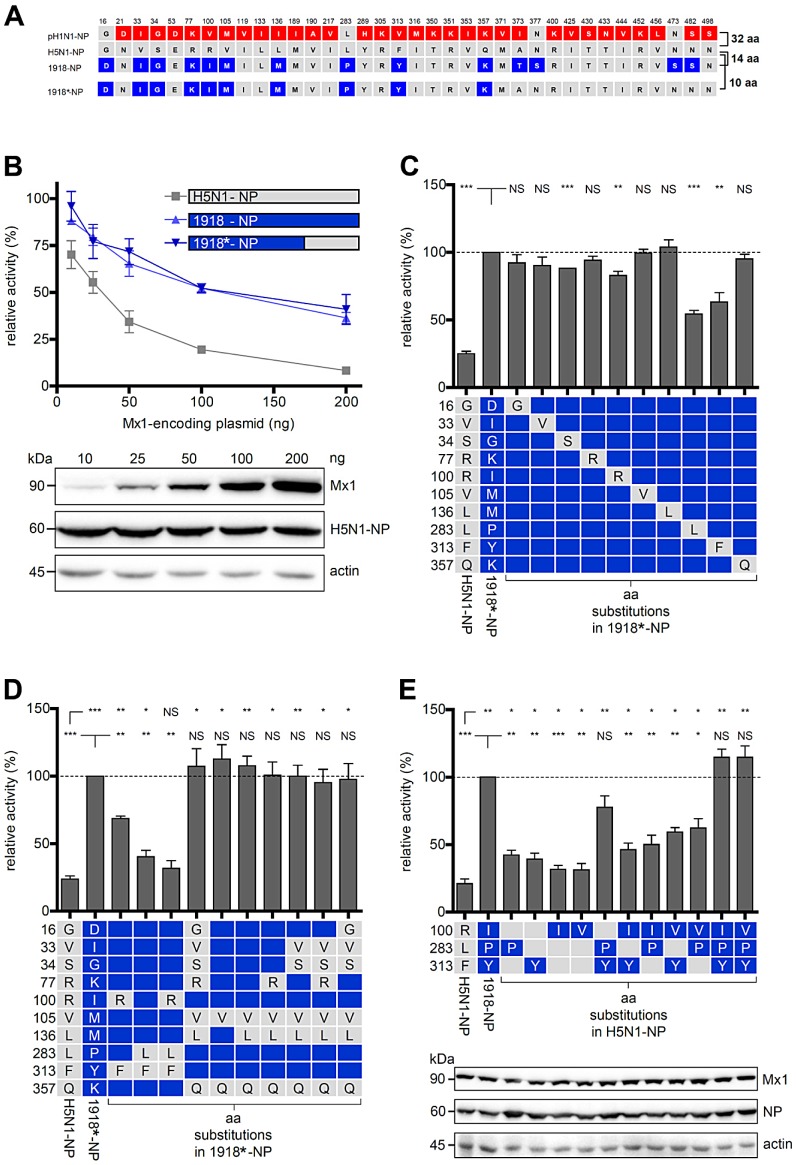
Identification of residues in the NP of the pandemic 1918 influenza A virus responsible for resistance to murine Mx1. (**A**) Amino acid differences between NP of H5N1, 1918 and pH1N1. Deviant amino acids of pH1N1 or 1918 NP are highlighted in red and blue, respectively. The 1918/H5N1 chimera (1918*-NP) comprises the N-terminal 365 amino acids of 1918 NP and the C-terminal 133 amino acids of the H5N1 NP and thus lacks 4 1918 NP-specific amino acids. (**B**) Viral polymerase activity in the presence of increasing concentrations of Mx1. HEK293T cells were transfected with expression plasmids coding for the PB2, PB1 and PA of H5N1, the indicated NP proteins, the firefly luciferase encoding minigenome, increasing amounts of Mx1-coding plasmid and a Renilla-expressing plasmid to normalize variation in transfection efficiency. Polymerase activity (relative activity) in the presence of antivirally inactive Mx1-K49A was used to normalize the data obtained with Mx1. Error bars indicate the standard error of the mean of three independent experiments. Western blot analysis was performed to determine the expression levels of Mx1 and H5N1 NP. (**C–E**) H5N1 polymerase activity was determined as in (B) after co-transfection of the expression plasmids coding for Mx1 (200 ng) and the indicated NP mutants (100 ng). The polymerase activity (relative activity) observed in the presence of Mx1 was normalized to Mx1-K49A. The resulting relative activity in the presence of either 1918*NP (C–D) or 1918 NP (E) was set to 100%. Western blot analysis shown in panel (E) was performed to determine the expression levels of NP. Error bars indicate the standard error of the mean of three independent experiments. Student's *t*-test was performed to determine the *P* value. *P<0.05, **P<0.01, ***P<0.001; NS, not significant.

We have previously shown that the polymerase activity of A/Thailand/1(KAN-1)/04 (H5N1) is highly sensitive to inhibition by murine Mx1, a close homolog of human MxA, in a polymerase reconstitution assay, and that this sensitivity is determined by the NP gene [21]. The H5N1 NP is of typical avian origin and resembles the avian H5N1 amino acid consensus sequence [21]. We therefore used this assay to identify the amino acids in NP of either the 1918 (A/Brevig Mission/1/1918) or the 2009 (A/Hamburg/4/2009) pandemic H1N1 strain (Figure 1A) critical for Mx1 resistance. In this assay, the polymerase activity was measured in the presence of overexpressed Mx1. In addition, we determined the polymerase activity in the presence of the inactive mutant Mx1-K49A [22]. Mx1 resistance was defined as the relative activity of the viral polymerase in the presence of Mx1 divided by the activity obtained with Mx1-K49A. Substitution of the H5N1 NP with the NP of the pandemic 1918 strain [23] rendered the H5N1 polymerase largely Mx1-resistant (Figure 1B). An alignment of the amino acid sequences of the 1918 NP with the NP of the Mx1-sensitive H5N1 strain revealed differences at 14 positions, including 4 positions in the C-terminal domain, namely amino acids 373, 377, 473 and 482 (Figure 1A). An artificial chimera (1918*-NP), consisting of the N-terminal 365 amino acids of the 1918 NP and the C-terminal domain (amino acids 366 to 498) of the H5N1 NP, behaved like the full-length 1918 NP, indicating that the 4 C-terminal differences in the 1918 protein do not contribute to the Mx1 resistance phenotype (Figure 1B). To investigate which of the 10 remaining 1918-specific amino acids in the chimeric protein contributed to Mx1 resistance, 1918*-NP mutants harboring single H5N1-derived substitutions were tested. A major decrease in Mx1 resistance was observed for the mutations P283L and Y313F, while a less pronounced phenotype was observed for I100R and several other mutants (Figure 1C). Various combinations of these putative adaptive mutations revealed that the triple mutant I100R, P283L, and Y313F led to a similar degree of Mx1 sensitivity as observed using the H5N1 NP (Figure 1D), whereas combinations of the remaining seven mutations failed to reduce Mx1 resistance (Figure 1D). Consistently, the 1918*-NP, carrying the mutations I100R, P283L and Y313F reduced Mx1 resistance by 50% (as compared to 1918*-NP) also in the context of the 1918 polymerase (Figure S1A–B).

To test whether the amino acids apparently responsible for Mx1 resistance of 1918 NP could also confer Mx1 resistance to an Mx1-sensitive NP, we introduced the mutations R100I, L283P, and F313Y into H5N1 NP. We also tested the exchange R100V in NP, since screening of the NCBI influenza database revealed that valine rather than isoleucine is commonly found at this position in seasonal strains [24]. Single amino acid exchanges slightly increased Mx1 resistance, while the combination of all three substitutions resulted in resistance comparable to the 1918 NP, irrespective of I or V at position 100 (Figure 1E). Importantly, this enhanced Mx1 resistance was not simply achieved by a higher polymerase activity, as it did not strictly correlate with increased activity in the presence of the antivirally inactive mutant Mx1-K49A. Nevertheless, some Mx1 resistance-enhancing amino acids appeared to improve the polymerase activity for unknown reasons in the absence of Mx1 or presence of Mx1-K49A protein (Figure S1C).

### The cluster of adaptive mutations conferring Mx1 resistance differs between pH1N1 and 1918 NP

**Figure 2:**
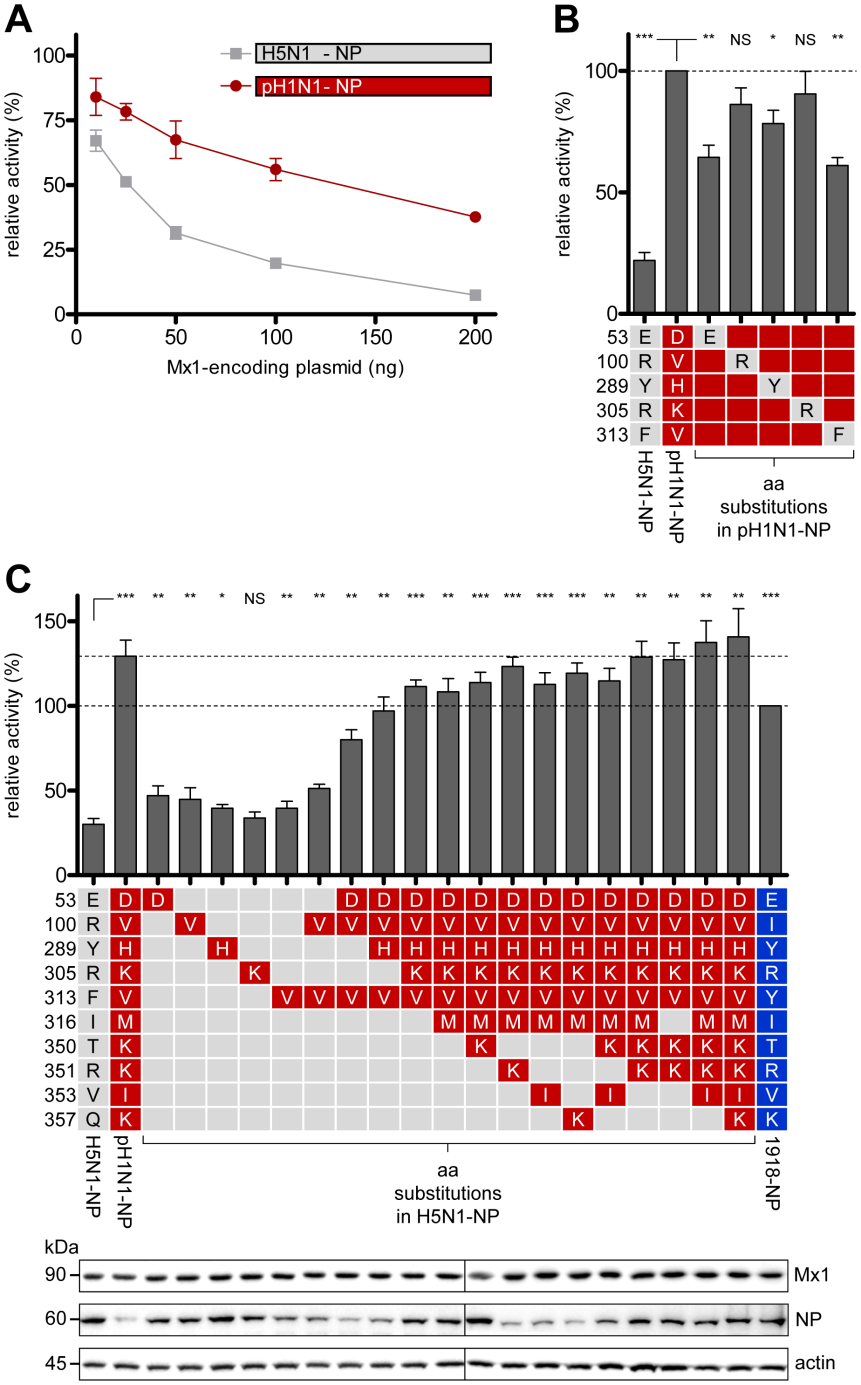
Identification of residues in the NP of the pandemic 2009 influenza A virus responsible for resistance to murine Mx1. (**A**) Reporter activity of H5N1 polymerase in HEK293T cells after co-transfection of expression plasmids coding for the indicated NP proteins (100 ng) and increasing amounts of Mx1. Polymerase activity in the presence of Mx1-K49A was used to normalize the data obtained with Mx1. Error bars indicate the standard error of the mean of three independent experiments. (**B**) H5N1 polymerase activity (relative activity) was determined as in (A) after co-transfection with expression plasmids coding for Mx1 (200 ng) and the indicated pH1N1 NP mutants (100 ng) harboring single H5N1-derived substitutions. The activity in the presence of Mx1 was normalized to the activity observed with the inactive Mx1 mutant Mx1-K49A. The activity observed in the presence of pH1N1 NP was set to 100%. Error bars indicate the standard error of the mean of three independent experiments. Student's *t*-test was performed to determine the *P* value. *P<0.05, **P<0.01, ***P<0.001; NS, not significant. (**C**) H5N1 polymerase activity was determined as in (B) after co-transfection with expression plasmids coding for Mx1 (200 ng) and the indicated H5N1-NP mutant proteins (100 ng) harboring single or multiple pH1N1-derived substitutions. The activity in the presence of Mx1 was normalized to the activity observed with the inactive Mx1 mutant Mx1-K49A. The activity observed in the presence of 1918 NP was set to 100%. Western blot analysis shown in the lower panel was performed to determine the expression levels of NP and Mx1. Error bars indicate the standard error of the mean of three independent experiments. Student's *t*-test was performed to determine the *P* value. *P<0.05, **P<0.01, ***P<0.001; NS, not significant.

Next, we evaluated the capacity of NP from the 2009 pandemic H1N1 influenza A virus (pH1N1) to confer Mx1 resistance in the context of the H5N1 polymerase. Figure 2A shows that pH1N1 NP rendered the H5N1 polymerase activity relatively resistant to Mx1 inhibition, as previously reported [21]. Sequence comparisons between the NP of pH1N1 and H5N1 origin revealed that pH1N1 NP carried only one (V100) out of the three Mx1 resistance determinants identified in 1918 NP (Figure 1A). We therefore assumed that different amino acids contribute to Mx1 resistance in pH1N1 NP than in 1918 NP. Since the pH1N1 NP differs by 32 amino acids from the H5N1 sequence (Figure 1A), we did not assay all individual amino acid positions, but rather focused on discordant and surface exposed amino acids in close proximity (27 Å) to the resistance cluster identified in the 1918 NP, utilizing the published NP crystal structures [25], [26]. Five of the resulting 10 pH1N1-specific amino acids, which were closest to the 1918 resistance cluster, were analyzed in the H5N1 polymerase reconstitution assay using pH1N1 NP mutants harboring single H5N1-derived substitutions (Figure 2B). A significant decrease in Mx1 resistance was observed for the mutations D53E, H289Y and V313F, while a less pronounced phenotype was observed for V100R and K305R (Figure 2B). Next, pH1N1-specific amino acids were introduced into H5N1 NP and tested for their contributions to Mx1 resistance in the H5N1 polymerase reconstitution assay (Figure 2C). While no individual amino acid substitution had a major effect, the combination of mutations at 4 positions (E53D, R100V, Y289H, and F313V) enhanced Mx1 resistance to a similar extent as 1918 NP. The additional mutations R305K, I316M, T350K, R351K, V353I, and Q357K together further increased Mx1 resistance to the degree of pH1N1 NP (Figure 2C). Although we observed variations in NP expression levels (Figure 2C), these differences did not correlate with Mx sensitivity. Together, these results demonstrate that the cluster of amino acids conferring Mx1 resistance differs between the NP of the 2009 and 1918 pandemic strains, although the crucial residues in both cases are located in the surface-exposed body domain.

### Adaptive mutations in NP confer resistance to both murine Mx1 and human MxA

**Figure 3:**
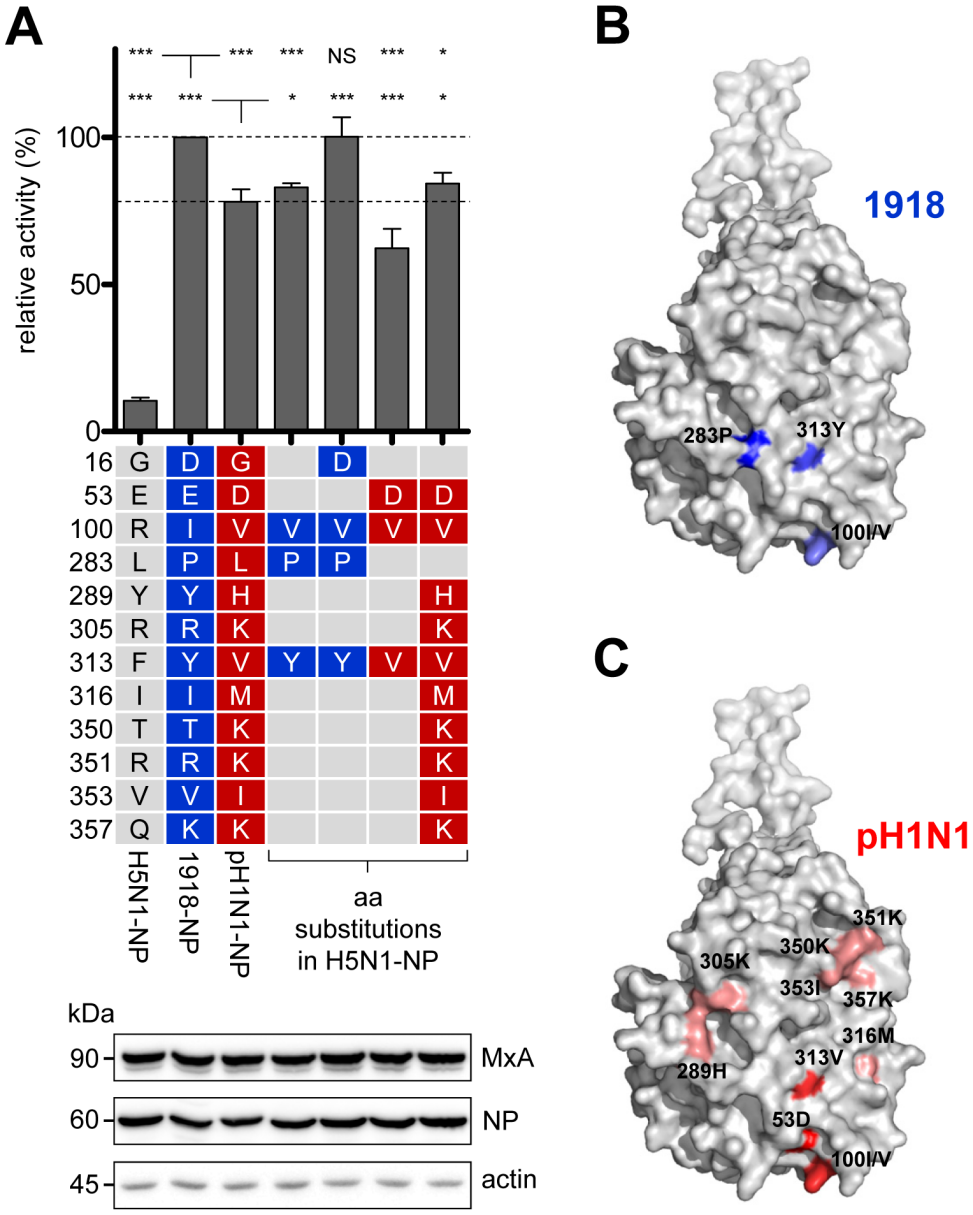
Amino acid clusters in NP of both the 1918 and pH1N1 strain mediate MxA resistance. (**A**) H5N1 polymerase activity in HEK293T cells after co-tranfection of the indicated expression plasmids coding for the NP mutants (100 ng) and MxA (200 ng). The activity in the presence of MxA was normalized to the activity observed with the inactive mutant MxA-T103A [66]. The activity observed in the presence of pH1N1 NP was set to 100%. Error bars indicate the standard error of the mean of three independent experiments. Student's *t*-test was performed to determine the *P* value. *P<0.05, ***P<0.001, NS, not significant. Western blot analysis was performed to determine the expression levels of MxA and the indicated NPs. (**B–C**) Amino acid positions of NP mediating Mx resistance. The program PyMOL was used to assign the indicated positions based on the structural model of A/HK/483/97(H5N1) NP (PDB code:2Q06). Positions of adaptive mutations required for Mx resistance of the 1918 NP are marked in blue (B). Amino acids of pH1N1 NP that exhibit only minor contribution to Mx resistance are highlighted in light red, whereas amino acids that strongly increased Mx resistance are indicated in red (C).

Next, we investigated whether the identified amino acid clusters in NP of the 1918 and the pH1N1 strains also confer resistance to human MxA. Consistent with the findings observed with Mx1, both 1918 and pH1N1 NP increased resistance in the H5N1 polymerase reconstitution assay (Figure 3A), however, the 1918 NP confers greater resistance to human MxA than to murine Mx1, whereas the opposite is true for the pH1N1 NP (Figure 2C and 3A). Importantly, the mutant H5N1 NP containing the 1918-derived Mx1 resistance determinants R100V, L283P, and F313Y exhibited an MxA resistance approximately 85% of that by 1918 NP itself (Figure 3A). To identify additional amino acid residues that contribute to the increased MxA resistance of the 1918 NP, we changed single amino acid positions in the 1918*-NP to avian residues. This revealed that D16, in addition to V100, P283, and Y313, contributed to MxA resistance (Figure S2). To confirm the relevance of this finding, we tested H5N1 NP harboring all four mutations (G16D, R100V, L283P and F313Y). This mutant NP displayed an MxA resistance comparable to 1918 NP (Figure 3A). Next, the pH1N1-specific adaptive mutations were tested in the context of H5N1 NP. In particular E53D, R100V and F313V increased resistance to MxA, while introduction of the additional mutations Y289H, R305K, I316M, T350K, R351K V353I and Q357K was required to achieve a resistance comparable to pH1N1-NP (Figure 3A). These results indicate that the adaptive mutations in 1918 or pH1N1 NP lead to increased resistance for both murine Mx1 and human MxA. Again, the observed resistance towards MxA did not strictly correlate with polymerase activity (Figure S3).

The atomic crystal structure of the H5N1 NP [26] revealed that the 1918-specific amino acids 100, 283 and 313 form a surface exposed cluster in the body domain of the viral NP (Figure 3B). Amino acid 16 is located in the N-terminal region of NP that is predicted to form a flexible loop adjacent to the 1918 cluster (Figure S4). The amino acids forming the pH1N1 cluster are located in the same area of the NP body domain as the 1918 cluster (Figure 3C).

### Positive selection of MxA resistance-enhancing NP mutations in the human host

**Figure 4:**
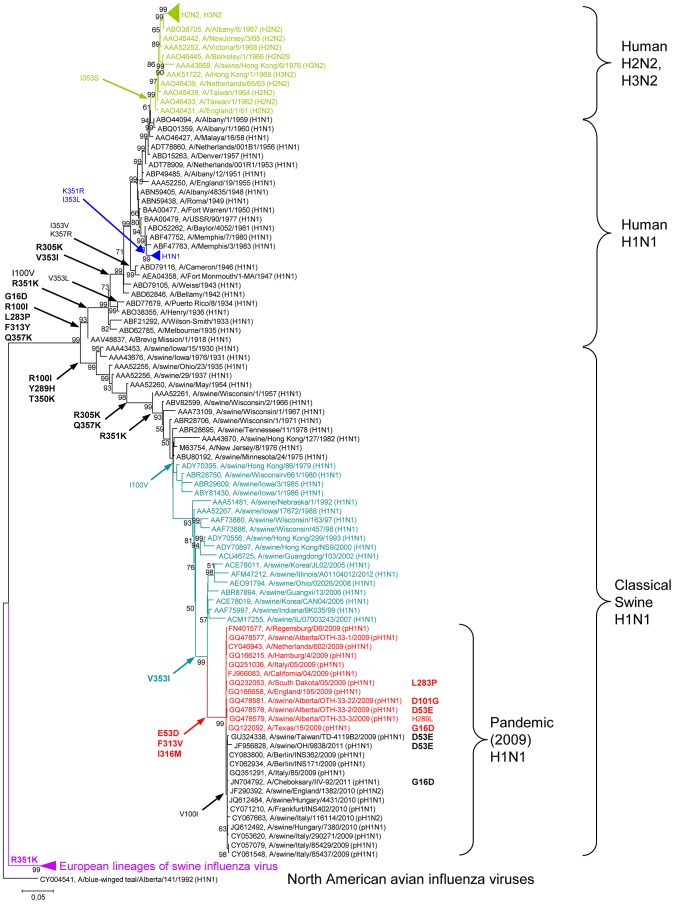
Phylogenetic analysis of representative NP sequences and the presence or loss of Mx-resistance enhancing mutations. The maximum likelihood tree of 147 aligned representative NP sequences shows four genotypes, i.e., (i) the human seasonal H1N1, H2N2 and H3N2 viruses, (ii) the classical swine H1N1 viruses and pandemic (2009) H1N1 viruses, (iii) the European lineages of swine influenza viruses, and (iv) the North American avian influenza viruses. Strain designations and GenBank acc. nos. are presented. Numbers at nodes indicate bootstrap values obtained after 1,000 replications. Only bootstrap values greater 50% were presented. The bar indicates substitutions per site. Three branches (H2N2/H3N2, recent human H1N1 strains, European lineages of swine influenza viruses) were condensed for clarity. The complete phylogenetic tree is shown in Figure S5. No relevant amino acid substitutions were observed in the condensed branches. Alterations of amino acid positions shown to influence Mx resistance (Figure 3, Figure S9) are highlighted in bold.

V100, P283, and V/Y313 mainly responsible for Mx1 or MxA resistance (Figure 3B and C), we analyzed the NP sequences of various isolates deposited in the NCBI Influenza Virus Sequence Database [24]. In avian isolates, each resistance-conferring amino acid could be identified in only ≤1% of the sequences (n = 5350) investigated (Table 1). In contrast, in classical seasonal human isolates representing H1N1, H1N2, H2N2, and H3N2 subtypes (n = 4969), the resistance-associated amino acids D16, I/V100, P283, and Y313 were each found at very high frequencies (>98%). Similarly, analyses of the human-derived pH1N1 NP sequences (n = 4104) revealed a high conservation of amino acids D53, I/V100 and V313 (>99%). Intriguingly, a chronological sequence comparison revealed that additional Mx resistance-enhancing mutations occurred in the NP of strains which are classified as descendents of the 1918 virus, namely R305K and R351K (Figure 4, Figure S5). These mutations emerged in early seasonal H1N1 viruses and were maintained in subsequent H2N2 and H3N2 strains (Figure 4). Taken together, these findings suggest a continuous selection pressure for increased Mx resistance in seasonal influenza viruses.

**Table 1:** Conservation of amino acid positions in NP that are responsible for MxA resistance.

Host	Subtypes	16D	53D	100I/V	283P	313V	313Y	n =
Avian	All	0.1	0.0	0.4	0.1	0.0	0.0	5350
Avian	H5N1	0.0	0.0	0.0	0.1	0.0	0.0	1130
Human	H5N1	0.0	0.0	0.5	0.5	0.0	0.0	187
Human	H1N1 seasonal	98.2	0.1	99.7	98.2	0.0	98.4	1383
Human	H1N2/H2N2/H3N2	99.5	0.0	99.8	99.5	0.0	99.4	3586
Swine	Classical H1N1	0.0	0.0	99.5	0.0	0.0	0.0	393
Human	pH1N1	0.3	100.0	99.9	0.0	99.8	0.0	4104
Swine	pH1N1	0.0	83.2	97.5	0.0	98.3	0.0	119

Full-length NP protein sequences of the indicated subtype and host were downloaded at 19th of October 2012 from [24]. Sequences depicted as H1N1 seasonal exclude H1N1 viruses of the pH1N1 lineage. Human pH1N1 refer to sequences of isolates found in humans. In avian sequences all available subtypes (H1-16, N1-9) were included. Sequences depicted as swine classical comprise sequences of the North American classical swine influenza viruses and exclude viruses of the pH1N1 lineage. The frequency of conserved residues is indicated in %. n = number of strains analyzed.

### NPs of the classical swine lineage confer partial resistance to MxA

**Figure 5:**
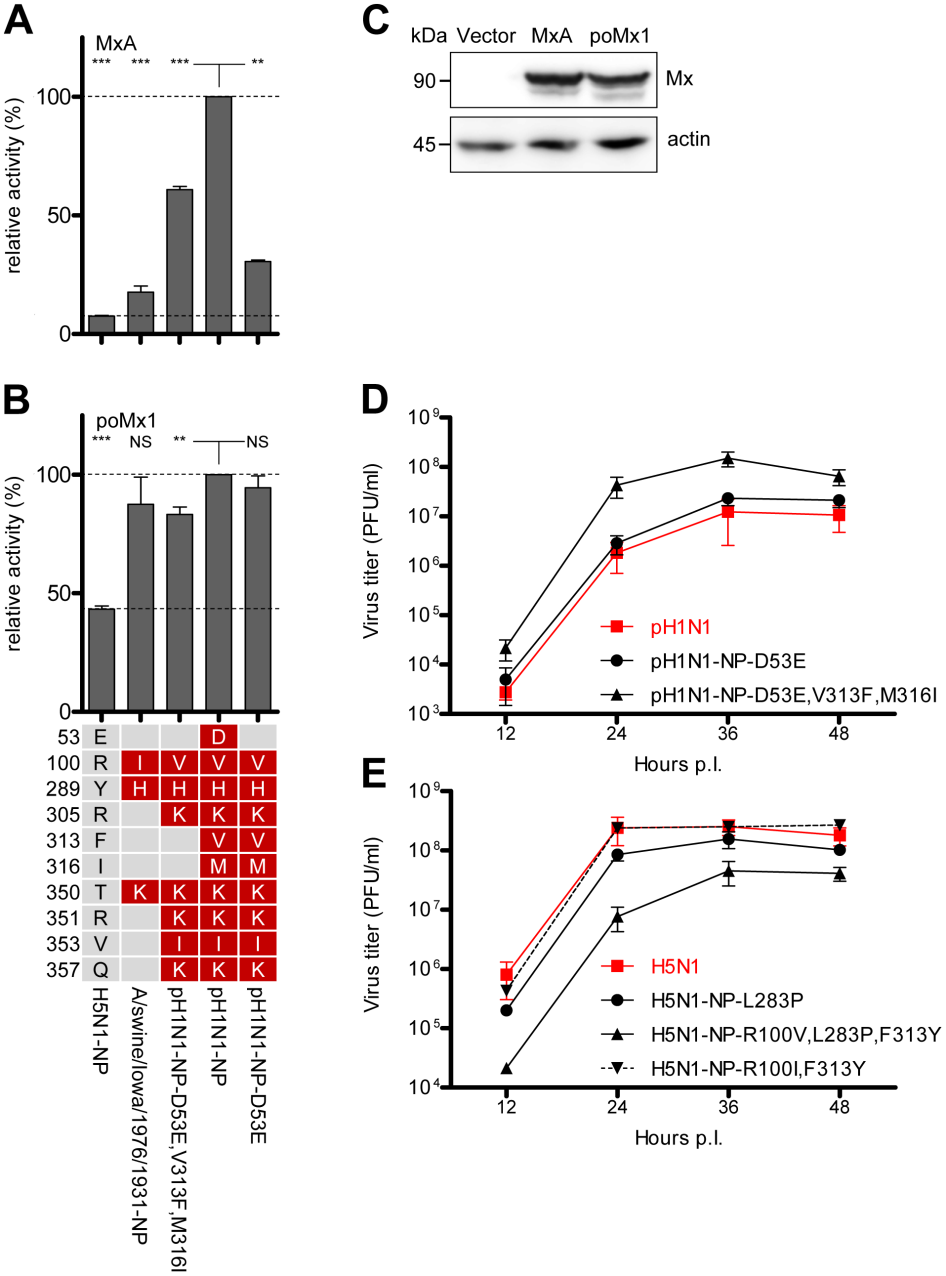
Mx resistance is accompanied by impaired viral growth in cell culture. (**A–B**) H5N1 polymerase activity in HEK293T cells after co-transfection of the indicated expression plasmids coding for NP (100 ng) and MxA (200 ng) (A) or porcine Mx1 (poMx1) (200 ng) (B). The activity in the presence of human MxA or poMx1 was normalized to the activity observed with the inactive mutant MxA-T103A. The activity observed with pH1N1 NP was set to 100%. Error bars indicate the standard error of the mean of three independent experiments. Student's *t*-test was performed to determine the *P* value. **P<0.01, ***P<0.001; NS, not significant. (**C**) Expression levels of human MxA and poMx1 in HEK293T cells after reconstitution of the H5N1 polymerase complex using an Mx-specific antibody. (**D–E**) MDCKII cells were infected with an MOI of 0.001 of wild-type or the indicated pH1N1 (D) or H5N1 mutant viruses (E) and incubated at 37°C. At the indicated time points post infection (p.i.), virus titers were determined by plaque assay. Error bars indicate the standard error of the mean of three independent experiments.

Since pH1N1 NP is derived from an influenza A virus of the classical swine lineage [10], [27], we analyzed the NP sequences of a number (n = 393) of corresponding swine isolates obtained between 1930 and 2012. Amino acids I/V100 were highly conserved (>99%), but D53 and V313 were not present in any of the NP sequences, which instead harbored the avian consensus amino acids at these positions (Table 1). We therefore anticipated that NPs of the classical swine influenza strains would confer less MxA resistance than pH1N1 NP. Indeed, NP of one of the first swine isolates such as A/swine/Iowa/1976/1931 displayed comparatively poor MxA resistance in the H5N1 polymerase reconstitution assay (Figure 5A). Importantly, NP of the classical swine influenza A virus lineage acquired the additional mutations 305K, 351K, 353I and 357K over time (Figure 4), resulting in a gradual increase in MxA resistance (Figure 5A, pH1N1-NP-D53E, V313F, M316I, third column from the left and Figure S6A). These changes, however, were not sufficient to confer MxA resistance comparable to that of pH1N1 NP. Interestingly, NP of the recent triple reassortant swine isolate A/swine/Ohio/02026/2008 (H1N1) and a hypothetical NP precursor of pH1N1 (pH1N1-NP-D53E, V313F, M316I) share the adaptive mutations 100I/V, 289H, 305K, 350K, 351K, 353I and 357K. The hypothetical NP precursor of pH1N1 was created by altering the human pH1N1 specific positions D53, V313 and M316 to the consensus found in classical swine H1N1 strains (D53E, V313F, M316I). These mutations are found at the branching point of classical swine influenza viruses and pH1N1 viruses (Figure 4). The hypothetical NP precursor conferred only partial MxA resistance in the H5N1 (Figure 5A and Figure S6A) as well as in the pH1N1 background (Figure S6C). These findings suggested that further adaptive mutations are needed which may affect MxA recognition or otherwise improve NP functions such as binding to viral (e.g PB2 [28] or cellular components (importins [13], helicases [29], [30]). Indeed, acquisition of E53D, F313V, and I316M was required to gain the full resistance of pH1N1 NP (Figure 5A and Figure S6C).

The antiviral potency of Mx proteins of pigs is still insufficiently characterized [31]–[33]. In the present reconstitution assay using HEK293T cells, porcine Mx1 (*Sus scrofa domestica*) decreased the H5N1 polymerase activity to 50% (Figure 5B), whereas human MxA reduced the activity to approximately 10% (Figure 5A), despite similar expression levels of both Mx proteins (Figure 5C). Likewise, the H5N1 polymerase activity in the presence of different NPs from several distinct swine isolates was not significantly affected by porcine Mx1 (Figure 5B, Figure S6B). To further test the antiviral strength of porcine Mx, we used porcine cells for the polymerase reconstitution assay. We found essentially the same extent of inhibition by the porcine Mx1 as in human 293T cells. As shown in Figure S7A, porcine Mx1 reduced the H5N1 polymerase activity to ca. 50% in swine NPTr or NSK cells [34]. In contrast, human MxA reduced the activity to 10% in porcine cells (Figure S7A). We conclude that the antiviral effect of porcine Mx1 is weak both in porcine and human cells. Together, these data suggest that while there is a clear selection pressure for swine influenza A viruses to acquire Mx resistance, the selection pressure in the porcine host is comparatively weak. Clearly, additional adaptive mutations are required to escape MxA restriction in humans.

### MxA resistance-enhancing mutations impair virus growth

Re-transmission of pH1N1 [35] from human to swine resulted in 17% of the documented cases in a substitution of aspartic acid at position 53 to the avian consensus glutamic acid (D53E) (swine pH1N1 in Table 1 and Figure 4), a mutation that confers loss of resistance to human MxA (Figure 5A), but not to porcine Mx1 (Figure 5B). This might suggest that MxA resistance-enhancing mutations are not necessarily favorable for NP function and might therefore cause impaired viral fitness.

To compare the replication fitness of viruses containing MxA-sensitive or MxA-resistant NPs, we infected MDCKII cells (which do not express antivirally active Mx proteins [36]) with pH1N1 or mutant viruses with enhanced MxA sensitivity. A recombinant pH1N1 virus with the single D53E reversion (pH1N1-NP-D53E) grew equally well as the parental pH1N1 virus (Figure 5D). In contrast, the pH1N1 precursor virus lacking three MxA resistance-enhancing mutations (pH1N1-NP-D53E,V313F,M316I) grew to approximately one log_10_ higher infectious titers throughout the course of infection (Figure 5D). These results demonstrate that reversions to the original amino acids of the putative swine precursor virus (Figure 4) provided a strong growth advantage in the absence of an antivirally active Mx. Thus, the acquisition of MxA resistance appears to cause some growth disadvantage.

To confirm this hypothesis, we tested the human H5N1 strain KAN-1 containing MxA resistance-enhancing mutations in MDCKII cells. Consistent with previous observations [36], the polymerase activity of H5N1 was not affected in the presence of canine Mx1 or Mx2 (Figure S7B). The triple mutant H5N1-NP-R100V,L283P,F313Y achieved reduced viral titers in the order of 1–2 log_10_ (Figure 5E). The recombinant H5N1 virus with the single MxA resistance mutation L283P (H5N1-NP-L283P) grew slightly less well than the parental H5N1 strain, while the double mutant virus H5N1-NP-R100I,F313Y showed comparable growth (Figure 5E). Intriguingly, the latter virus showed severely impaired replication efficiency in avian LMH cells which lack antiviral Mx proteins [37]–[39] (Figure S8). We conclude that MxA resistance is linked to impaired viral growth and may be easily lost in the absence of selective pressure.

### Mx resistance-enhancing mutations in NP increase the virulence of the H5N1 strain KAN-1 in Mx1-positive mice

**Figure 6:**
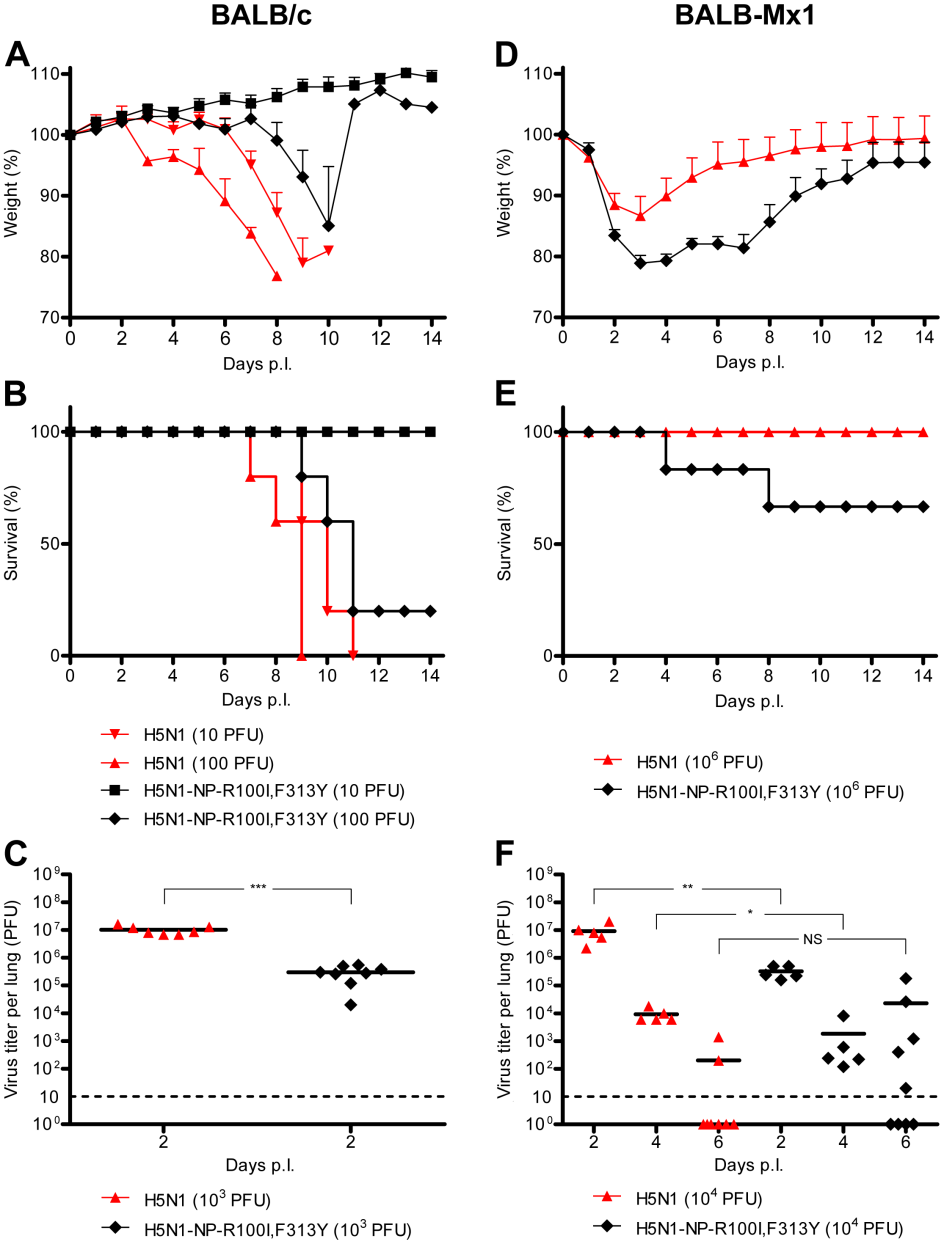
Mx resistance-enhancing mutations in NP increase the virulence of the H5N1 isolate KAN-1 in Mx1-positive mice. (**A–C**) BALB/c mice were inoculated intranasally with the indicated amount of viruses. Changes in body weight (A) or survival (B) (*n* = 6/group) were monitored daily for 14 days. Lungs from infected mice were collected 2 days p.i., homogenized and virus titers were determined by plaque assay (C). (**D–F**) Mx1-positive BALB/c mice were inoculated intranasally with the indicated amount of viruses. Changes in body weight (D) or survival (E) (*n* = 6/group) were monitored daily for 14 days. Lungs from infected mice were collected 2, 4, and 6 days p.i., homogenized and virus titers were determined by plaque assay (F). Student's *t*-test was performed to determine the *P* value. *P<0.05, **P<0.01, ***P<0.001; NS, not significant.

We argued that the acquisition of Mx resistance should also increase the pathogenicity of H5N1 in Mx1-positive mice. To test this hypothesis we selected the double mutant virus H5N1-NP-R100I,F313Y which had almost wild-type growth characteristics in tissue culture (Figure 5E) and exhibited comparable polymerase activity (Figure S8A), in spite of its Mx resistance-enhancing mutations (Figure 1E). We could not include the triple mutant virus H5N1-NP-R100V,L283P,F313Y in our studies due to the emergence of escape mutants (data not shown) and its strong attenuation. First, we compared the growth of wild-type and mutant H5N1 viruses in Mx1-negative BALB/c mice. Infection with 10 PFU of wild-type H5N1 virus lead to pronounced weight loss and death of all animals, as expected [40]. In contrast, infection with the same challenge dose (10 PFU) of mutant H5N1-NP-R100I,F313Y virus resulted in survival of all BALB/c mice without significant weight loss (Figure 6A and B), indicating that the two amino acid substitutions associated with Mx resistance caused impaired viral growth. Indeed, challenge of BALB/c mice with 1000 PFU of the mutant virus resulted in viral lung titers that were 15-fold reduced as compared to wild-type virus at 48 h after infection (Figure 6C). Next, the growth properties of the two viruses were studied in congenic Mx1-positive mice. In these animals, infection with wild-type H5N1 virus produced no pronounced pathological effects, even at high doses of 10^6^ PFU (Figure 6D and E). In contrast, Mx1-positive mice showed significant weight loss and mortality when challenged with 10^6^ PFU of mutant H5N1-NP-R100I,F313Y virus (Figure 6D and E). To assess viral growth in Mx1-positive mice, viral lung titers were determined at various time points after intranasal infection with 10^4^ PFU (Figure 6F). Two days after infection, the titers in mice infected with the mutant virus were approximately 28-fold lower than those in mice infected with the wild-type virus, demonstrating the attenuating effect of the Mx resistance-enhancing mutations in NP. Four days after infection, a 5-fold difference was observed, and 6 days after infection the mutant virus was still present in 5 out of 9 Mx1-positive mice with titers up to 2×10^5^ PFU. In contrast, only low titers of wild-type virus were detected at the same time in 2 out of 9 animals. We conclude from these experiments that H5N1 viruses harboring MxA resistance-enhancing mutations partially overcome the antiviral effect mediated by Mx1.

## Discussion

**Figure 7:**
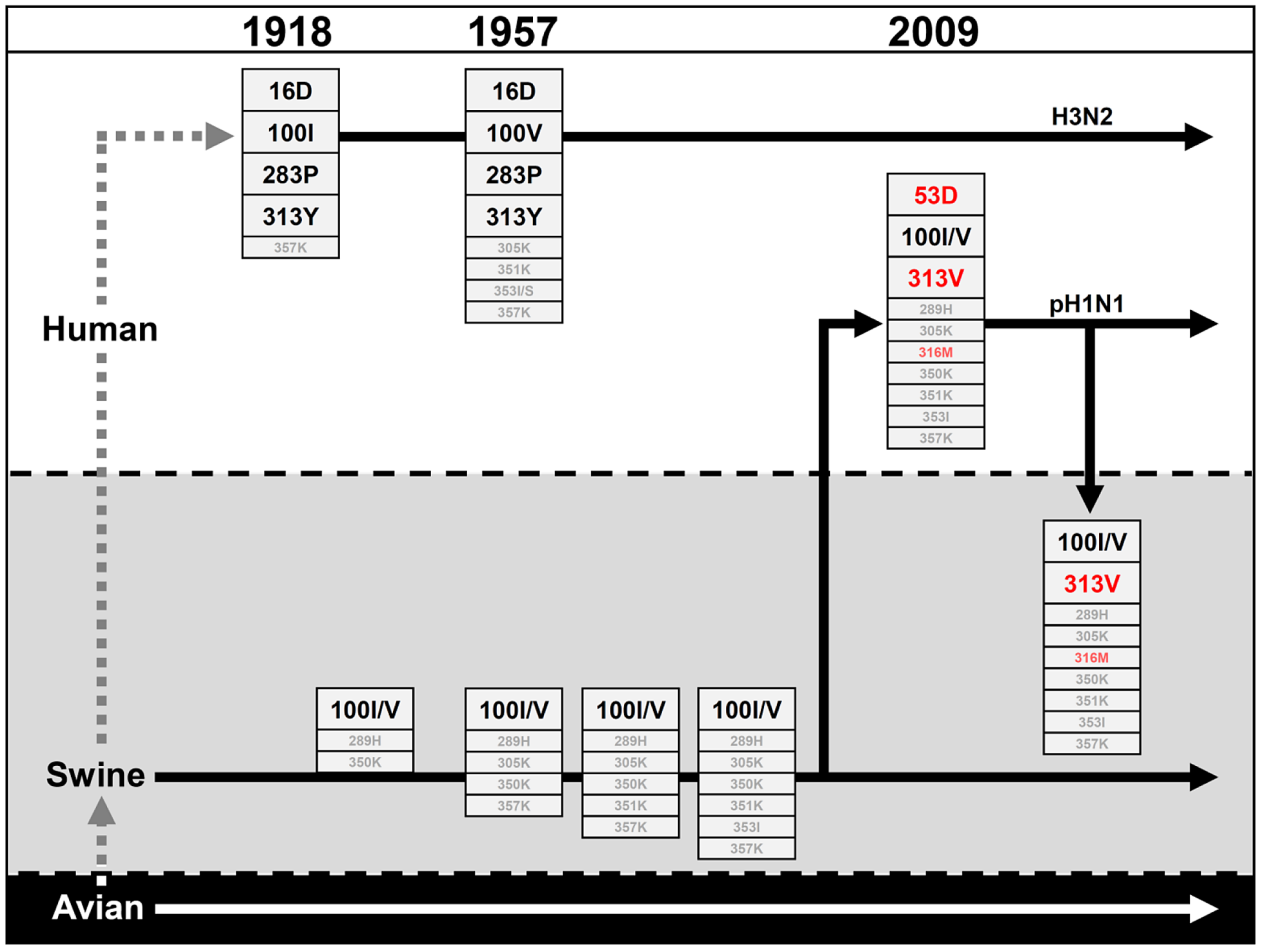
Temporal appearance of MxA resistance enhancing amino acids in NP of human and swine influenza A viruses. Bold letters indicate amino acids in NP shown to increase significantly MxA resistance, whereas amino acids highlighted in grey are minor contributors. Adaptive mutations that newly emerged with the appearance of the 2009 pandemic pH1N1 strain are depicted in red. 53D got partially lost after re-introduction of pH1N1 into the swine host.

Influenza A viruses sporadically transmit from the avian reservoir into the human population. Here we describe specific mutations found in the NP of the 1918 and 2009 pandemic viruses that confer resistance to the IFN-induced human MxA GTPase, a major restriction factor for influenza and other orthomyxoviruses. As MxA strongly inhibits transcription and replication of the viral genome early in infection, its antiviral activity can be readily analyzed in polymerase reconstitution (minireplicon) assays [20], [21]. Using this assay, we identified a cluster of surface-exposed amino acids in the body domain of NP crucial for Mx resistance. Interestingly, different amino acid positions were identified in 1918 and pH1N1 NP, yet all were located in the same domain. All resistance-associated amino acids are conserved in previous and current human influenza A viruses (Table 1), and the continuing acquisition of resistance-enhancing mutations (Figure 7) suggests strong positive selection pressure by MxA. Of note, mutations conferring MxA resistance are absent in avian influenza A viruses, although we did observe the emergence of adaptive NP mutations in avian-derived viruses circulating in swine (Figure 4 and Figure 7). These substitutions in NP not only increased resistance to swine Mx1 but also to human MxA, supporting the theory that swine are an excellent intermediate host for the generation of viruses with pandemic potential.

Influenza A viruses carrying a novel NP gene were introduced into the human population in 1918 and 2009 [10], [27], but the distinct MxA resistance clusters in the NP genes of these pandemic viruses suggest independent evolution (Figure 7). In case of the 2009 pH1N1 virus, the NP gene originated from the classical swine lineage, which itself is of avian origin [41]. Our data suggest that the swine precursor virus of pH1N1 acquired additional amino acid changes, which together increased the ability to counteract human MxA. The evolution of the 1918 NP is comparatively less clear. Recent data suggest that both the 1918 and the classical swine virus lineage share a common avian ancestor [9]. It is unresolved whether the avian precursor virus was first transmitted to humans and then onto swine or vice versa [9]. In the latter case, the precursor virus may have first adapted to swine Mx1 through the mutation R100I in NP which is found in early swine isolates [24]. In the first case, mutations at position 283 and 313 might have been lost after transmission from humans to swine, as observed with the pH1N1 virus. However, analyses of host specificity markers which discriminate human from avian influenza viruses indicate that four adaptive mutations in NP (16D, 283P, 313Y and 357K) were likely required for transmission of the 1918 precursor virus to humans [42]. Remarkably, all of these mutations contribute to MxA resistance (Figure 3) and may have evolved in a pre-pandemic phase in the human population. Since circulating human influenza A virus strains maintain these adaptive mutations (Table 1), it is conceivable that viruses are under constant selection pressure mediated by MxA.

We observed that the acquisition of Mx resistance had a negative effect on viral growth in the absence of MxA. When MxA resistance-enhancing mutations were introduced into highly pathogenic avian H5N1 viruses, the recombinant viruses grew less well than the wild-type H5N1 virus in MxA-negative MDCKII cells (Figure 5E), in BALB/c mice (Figure 6C) and even in avian cells (Figure S8). These mutations have no major effect in the viral polymerase reconstitution assay (Figure S1C and S3), and it is unclear which step of the viral replication cycle is attenuated in infected cells. If MxA resistance-associated amino acids are also counter-selected in circulating avian influenza strains, then the emergence of MxA resistance in the avian reservoir is expected to be an extremely rare event (Table 1). Of note, human H5N1 isolates have developed few if any of the identified MxA resistance-enhancing mutations (Table 1), most likely due to the associated strong attenuation [43] (Figure 5E, 6A–C). Perhaps for this reason, the 1957 and 1968 pandemic viruses retained the well-adapted 1918-origin NP, despite acquiring other avian genome segments by reassortment. Thus, more than 90 years passed before a new NP lineage was established 2009 in humans, a process aided by gradual adaptation of the new NP in swine.

Correspondingly, we observed an increase in viral growth of pH1N1 lacking the MxA resistance-enhancing mutations D53, V313 and M316 in MDCKII cells (Figure 5D) which do not express antivirally active Mx proteins [36]. We therefore propose that passage of viruses in hosts with weak or inactive Mx proteins (such as swine or laboratory mouse strains, respectively) would result in a loss of MxA resistance. In fact, experimental adaptation of the pandemic 2009 virus to Mx1-negative mice led to the acquisition of mutations in NP which strongly diminished Mx resistance, such as D101G [44] (Figure S9). Similarly, mutations conferring MxA resistance were lost (D53E, D101G, H289L) following re-transmission of pH1N1 from human to swine (Figure 4).

The mechanism by which MxA exerts its antiviral function during infection or in the polymerase reconstitution assays is presently not known. MxA may block the viral life cycle at several early steps by interfering with the processes of vRNP entry and intracellular transport [45], as well as primary [45] and secondary transcription [46]. We proposed a model in which MxA recognizes vRNPs and begins to self-assemble into rings, thereby sterically inhibiting vRNP function [15], [18], [19]. This model has recently been suggested also for mouse Mx1, but in a modified version involving in addition also the polymerase subunit PB2 [28], in agreement with previous functional work implicating PB2 as putative target of mouse Mx1 [47], [48]. Modeling the present MxA resistance clusters into the available vRNP structure [49] revealed that the sites on NP are most likely solvent-exposed, and thus accessible to cellular factors. Initial contact of MxA to single binding sites on NP might be weak but reinforced by oligomerization, involving multiple repetitive contacts exposed on the many NP molecules that form the vRNP. Weak but extensive contacts to repetitive viral target motifs have been demonstrated for other intracellular restriction factors. For example, TRIM5α specifically binds to several surface-exposed amino acids of the capsid protein of HIV-1, thereby forming an array or lattice on top of the viral capsid [50], [51]. To date, physical interaction between influenza A virus NP and MxA could be demonstrated after covalent protein crosslinking [52] and thus MxA might also bind to free NP in the cytoplasm thereby blocking polymerase activity indirectly. However, it is also likely that further cellular proteins modulate MxA activity and its interaction with viral proteins. Previous work identified a number of potential MxA co-factors, but their contribution is still unclear [53]–[56]. One promising candidate is the helicase UAP56, a DEAD box RNA helicase which was shown to prevent double-strand RNA formation and subsequent innate immune activation in influenza virus-infected cells. UAP56 binds both MxA and NP [29], [57], in the latter case via the N-terminal domain of NP which contains the MxA resistance-enhancing mutation G16D [58]. Nonetheless, the significance of this observation and the role of UAP56 for antiviral activity remain to be demonstrated.

In summary, we have found functional and evolutionary evidence that the human MxA GTPase provides an efficient barrier against zoonotic introduction of influenza A viruses into the human population. Thus, the human MxA is a significant driving force in influenza A virus nucleoprotein evolution. We therefore propose that amino acids known to contribute to MxA resistance should be monitored as a strong indicator for the pandemic potential of newly emerging influenza A viruses.

## Materials and Methods

### Ethics statement

All animal experiments were performed in compliance with the German animal protection law (TierSchG). The mice were housed and handled in accordance with good animal practice as defined by FELASA (www.felasa.eu/guidelines.php) and the national animal welfare body GV-SOLAS (www.gv-solas.de/index.html). The animal welfare committees of the university of Freiburg, as well as the local authorities (Regierungspräsidium Freiburg) approved all animal experiments.

### Cells

Canine MDCKII, porcine NSK and NPTr cells [34], and human HEK 293T cells were maintained in Dulbecco's modified Eagle's medium (DMEM) supplemented with 10% fetal calf serum, 2 mM L-glutamine and 1% penicillin-streptomycin. Chicken hepatocellular epithelial cell line (LMH) [59] was grown in DMEM supplemented with 8% fetal calf serum 2% chicken serum, 2 mM L-glutamine and 1% penicillin/streptomycin.

### Plasmid constructions

The pHW2000 rescue plasmids [60] and pCAGGS expression plasmids [61] coding for NP were used for site directed mutagenesis. The coding region of MxA was cloned into pCAGGS, whereas murine Mx1 was expressed using pcDNA 3.1 [21]. The cDNA of porcine Mx1 (poMx1) corresponding to the full length 1992 nt long open reading frame as described in [62], encoding a Flag tag at its 5′-end was cloned into pCAGGS using KpnI and XhoI. poMx1 cDNA was generated from mRNA isolated from IFNα-2a-treated cell cultures from domestic pig (*Sus scrofa domestica*).

### Generation of recombinant influenza A viruses

The recombinant viruses A/Hamburg/4/09 (pH1N1) and A/Thailand/1(KAN-1)/04 (H5N1), and the NP-mutant viruses were generated by the eight-plasmid reverse-genetics system as described previously [21]. All recombinant viruses were plaque purified on MDCKII cells. Virus stocks were prepared on MDCKII cells and titers were determined by plaque assay.

### Reconstitution of the influenza virus polymerase (minireplicon)

HEK 293T cells seeded in 12-well plates were transfected using the Nanofectin transfection reagent (PAA Laboratories) according to the manufacturer's protocol. 10 ng of pCAGGS plasmids encoding PB2, PB1, and PA and 100 ng of NP-encoding plasmid were cotransfected with 100 ng of the firefly luciferase- encoding viral minigenome construct pPolI-FFLuc-RT, which is flanked by the noncoding regions of segment 8 of influenza A virus. The transfection mixture also contained 30 ng of pRL-SV40, a plasmid constitutively expressing *Renilla* luciferase under the control of the simian virus 40 promoter to normalize variations in transfection efficiency. To evaluate the antiviral potential of Mx1 and MxA, we cotransfected Mx1- or MxA- encoding plasmid. A simultaneous experiment with cotransfection of the antivirally inactive mutants Mx1-K49A or MxA-T103A, respectively, was used as a control. To achieve equal amounts of transfected DNA, an empty vector plasmid was added. Twenty-four hours post transfection, cells were lysed and firefly and Renilla luciferase activities were measured using the dual luciferase reporter assay (Promega) according to the manufacturer's protocol. Reconstitution of the viral polymerase complex in avian and porcine cells was performed as above with the exception that the minigenome RNA was expressed under the control of a chicken [63] or porcine Pol I promoter (pSPOM2) [34].

### Virus growth curves

MDCKII cells seeded in 6-well plates were incubated with virus at a multiplicity of infection (MOI) of 0.001 in PBS^+/+^ containing 0.2% BSA for 1 h at 37°C. The inoculum was removed and 3 ml infection medium (DMEM supplemented with 0.2% BSA), additionally containing 1 µg/ml TPCK-treated trypsin for pH1N1 viruses, was added. Virus titers in cell culture supernatants were determined at the indicated time points by plaque assay and are expressed as PFU per ml.

### Primer extension analysis

For determination of viral transcript levels in virus-infected MDCKII cells, cells were seeded in 6-well plates and infection was carried out with infection media. After the indicated time point post infection, cells were harvested in Trizol™ and RNA was purified according to the manufacturer's protocol (Invitrogen). Primer extension analysis was performed as described [63] using specific primers for the NA segment (mRNA, cRNA and vRNA) and cellular 5sRNA.

### Animal experiments

BALB/c mice were obtained from Janvier (Straßburg) and congenic BALB.A2G-*Mx1* mice (designated BALB-Mx1) carrying the functional *Mx1* allele [64] were bred locally. Six- to eight-week-old mice were anesthetized with a mixture of ketamine (100 µg per gram body weight) and xylazine (5 µg per gram) administered intraperitoneally (i.p.) and inoculated intranasally (i.n.) with the indicated doses of viruses in 50 µl phosphate-buffered saline (PBS) containing 0.2% bovine serum albumin (BSA). Mice were monitored daily for weight loss until 14 days postinfection (p.i.). Animals with severe symptoms or more than 25% weight loss were euthanized. Lung homogenates were prepared using the FastPrep24 system (MP Biomedicals). Briefly, after addition of 800 µl of PBS containing 0.2% BSA, lungs were subjected to two rounds of mechanical treatment for 10 s each at 6.5 m/s. Tissue debris was removed by low-speed centrifugation. The LD_50_ values were calculated based on the infectious dose (PFU). All animal work was conducted under BSL 3 conditions in accordance with the guidelines of the local animal care committee.

### Molecular modeling

The program PyMOL (www.pymol.org) was used to assign the indicated positions in the structural model of the NP of A/HK/483/97(H5N1) (PDB code:2Q06). The program I-TASSER (zhanglab.ccmb.med.umich.edu/I-TASSER) was used to generate a full length NP model of A/Thailand/1(KAN-1)/04 (H5N1), including amino acids 1–20.

### Alignments and phylogenetic analyses

Alignments and phylogenetic analyses were conducted with MEGA5 [65]. For maximum likelihood (ML) tree inference, the GTR substitution model assuming gamma distribution (four gamma categories) and invariant sites was selected, and the initial tree was made automatically. Bootstrap analysis was performed with 1,000 replications. The optimal substitution model was selected on the basis of the Bayesian information criterion (BIC) and the corrected Akaike information criterion (AICc) using a model test implemented in MEGA5.

## Supporting Information

Figure S1 **Polymerase activities in the presence of Mx1 or the antivirally inactive mutant Mx1-K49A.** (**A**) Reporter activity of 1918 polymerase. HEK293T cells were transfected with expression plasmids coding for PB2, PB1 and PA of the pandemic 1918 strain, the indicated NP proteins, the firefly luciferase encoding minigenome, 200 ng Mx1-encoding plasmid and a *Renilla*-expressing plasmid to normalize variation in transfection efficiency. Polymerase activity in the presence of antivirally inactive Mx1-K49A was used to normalize the data obtained with Mx1. Activity in the presence of the 1918*-NP was set to 100%. Error bars indicate the standard error of the mean of three independent experiments. Student's *t*-test was performed to determine the *P* value. **P<0.01. (**B**) 1918 polymerase activity in the presence of the antivirally inactive mutant Mx1-K49A. HEK293T cells were transiently transfected with expression plasmids coding for the vRNP components as described in (A) including 200 ng of Mx1-K49A-encoding plasmid. Renilla activity was used to normalize variation in transfection efficiency. The polymerase activity in the presence of the 1918*-NP was set to 100%. Error bars indicate the standard error of the mean of three independent experiments. Student's *t*-test was performed to determine the *P* value. NS, not significant. (**C**) H5N1 polymerase activity in the presence of either Mx1, the antivirally inactive mutant Mx1-K49A or empty vector. HEK293T cells were transiently transfected with expression plasmids coding for the vRNP components of H5N1 including 200 ng of Mx1, Mx1-K49A-encoding plasmid or empty vector and the indicated NP mutants. Renilla activity was used to normalize variation in transfection efficiency. The polymerase activity in the presence of the 1918-NP was set to 100%. Error bars indicate the standard error of the mean of three independent experiments. Student's *t*-test was performed to determine the *P* value. *P<0.05, **P<0.01, ***P<0.001; NS, not significant.
(PDF)

Figure S2 **Identification of amino acids in 1918 NP responsible for MxA resistance.** Reporter activity of the H5N1 polymerase in HEK293T cells after co-transfection of the expression plasmids coding for MxA (200 ng) and the indicated NP proteins (100 ng). The activity in the presence of MxA was normalized to the activity observed with the antivirally inactive mutant MxA-T103A. The activity observed with the 1918*-NP was set to 100%.
(PDF)

Figure S3 **Polymerase activities in the presence of the antivirally inactive mutant MxA-T103A.** H5N1 polymerase reporter activity was determined after co-transfection of expression plasmids coding for the indicated NP mutants (100 ng) and the antivirally inactive mutant MxA-T103A (200 ng). The reporter activity observed with the 1918-NP was set to 100%. Error bars indicate the standard error of the mean of three independent experiments. Student's *t*-test was performed to determine the *P* value. *P<0.05, **P<0.01; NS, not significant.
(PDF)

Figure S4 **Localization of the adaptive mutation 16D in NP.** The model for the full-length structure of H5N1 NP (A/Thailand/1(KAN-1)/04) harboring the mutations G16D, R100I, L283P and F313Y was generated utilizing I-TASSER. The N-terminus that was not resolved in the crystal structure (aa 1–20) is highlighted in light orange, whereas 1918-specific amino acids that confer Mx resistance are shown in blue (16D, 100I/V, 283P and 313Y).
(PDF)

Figure S5 **Phylogenetic analysis of representative NP sequences and the presence or loss of Mx-resistance enhancing mutations.** The maximum likelihood tree of 147 aligned sequences shows four genotypes, i.e., (i) the human H1N1, H2N2 and H3N2 viruses, (ii) the classical swine H1N1 viruses and pandemic (2009) H1N1 viruses, (iii) the European lineages of swine influenza viruses, and (iv) the North American avian influenza viruses. Strain designations and GenBank acc. nos. are presented. Numbers at nodes indicate bootstrap values obtained after 1,000 replications. Only bootstrap values greater than 50% are presented. The bar indicates substitutions per site. Alterations of amino acid positions shown to influence Mx resistance (Figure 3, Figure S9) are highlighted in bold.
(PDF)

Figure S6 **Mx resistance of NP variants of swine origin.** (**A–B**) H5N1 or (**C**) pH1N1 polymerase activity in the presence of human MxA (A and C) or porcine Mx1 (poMx1) (B). The activity in the presence of human MxA or poMx1 was normalized to the activity observed after co-expression of the antivirally inactive MxA mutant MxA-T103A. The activity observed with the pH1N1-NP was set to 100%, respectively. Error bars indicate the standard error of the mean of three independent experiments. Student's *t*-test was performed to determine the *P* value. **P<0.01, ***P<0.001; NS, not significant.
(PDF)

Figure S7 **Inhibition of polymerase activities by porcine and canine Mx proteins.** (**A**) Swine NPTr or NSK cells were transfected with expression plasmids coding for NP, PB2, PB1 and PA of H5N1, the porcine Pol1-driven firefly luciferase encoding minigenome, 200 ng of either porcine Mx1 (poMx1), MxA, or the GTPase-inactive MxA mutant MxA-T103A and a *Renilla*-expressing plasmid to normalize variation in transfection efficiency. The activity in the presence of MxA-T103A was set to 100%. Error bars indicate the standard error of the mean of three independent experiments. Student's *t*-test was performed to determine the *P* value. **P<0.01; ***P<0.001. (**B**) HEK293T cells were transfected with expression plasmids coding for NP, PB2, PB1 and PA of H5N1, the firefly luciferase encoding minigenome, 200 ng of either canine Mx1 (cMx1), canine Mx2 (cMx2), MxA or the GTPase-inactive MxA mutant MxA-T103A and a *Renilla*-expressing plasmid to normalize variation in transfection efficiency. The activity in the presence of MxA-T103A was set to 100%. Error bars indicate the standard error of the mean of three independent experiments. Student's *t*-test was performed to determine the *P* value. ***P<0.001; NS, not significant.
(PDF)

Figure S8 **Mx resistance-enhancing mutations influence transcription and viral growth in avian cells.** (**A**) Comparison of viral transcription in MDCKII or avian LMH cells infected with an MOI of 5 of either H5N1 (KAN-1) or H5N1-NP-R100I,F313Y after the indicated hours post infection (h.p.i.). mRNA, cRNA, and vRNA levels were determined using primer extension analysis with primers specific for segment 6. Levels of cellular 5sRNA served as internal control. (**B**) Avian LMH cells were infected with an MOI of 0.001 of either H5N1 or H5N1-NP-R100I,F313Y and incubated at 37°C. At the indicated time points post infection (p.i.), virus titers were determined by plaque assay. Error bars indicate the standard error of the mean of three independent experiments.
(PDF)

Figure S9 **Mutation D101G in NP of pH1N1 strongly reduces resistance to MxA.** H5N1 polymerase activity after co-transfection of MxA (200 ng) and the indicated NP (100 ng) expression plasmids. The activity in the presence of MxA was normalized to the activity observed with the antivirally inactive mutant MxA-T103A. The reporter activity observed with the pH1N1-NP in the presence of MxA was set to 100%. Error bars indicate the standard error of the mean of two-three independent experiments.
(PDF)
