# Supplementary material for: Pandemic Influenza A Viruses Escape from Restriction by Human MxA through Adaptive Mutations in the Nucleoprotein
Source: PLoS Pathog. 2013 Mar 28;9(3):e1003279. doi: 10.1371/journal.ppat.1003279 (PMC3610643; doi:10.1371/journal.ppat.1003279)
Supplement: Figure S1 — Polymerase activities in the presence of Mx1 or the antivirally inactive mutant Mx1-K49A. (A) Reporter activity of 1918 polymerase. HEK293T cells were transfected with expression plasmids coding for PB2, PB1 and PA of the pandemic 1918 strain, the indicated NP proteins, the firefly luciferase encoding minigenome, 200 ng Mx1-encoding plasmid and a Renilla-expressing plasmid to normalize variation in transfection efficiency. Polymerase activity in the presence of antivirally inactive Mx1-K49A was used to normalize the data obtained with Mx1. Activity in the presence of the 1918*-NP was set to 100%. Error bars indicate the standard error of the mean of three independent experiments. Student's t-test was performed to determine the P value. **P<0.01. (B) 1918 polymerase activity in the presence of the antivirally inactive mutant Mx1-K49A. HEK293T cells were transiently transfected with expression plasmids coding for the vRNP components as described in (A) including 200 ng of Mx1-K49A-encoding plasmid. Renilla activity was used to normalize variation in transfection efficiency. The polymerase activity in the presence of the 1918*-NP was set to 100%. Error bars indicate the standard error of the mean of three independent experiments. Student's t-test was performed to determine the P value. NS, not significant. (C) H5N1 polymerase activity in the presence of either Mx1, the antivirally inactive mutant Mx1-K49A or empty vector. HEK293T cells were transiently transfected with expression plasmids coding for the vRNP components of H5N1 including 200 ng of Mx1, Mx1-K49A-encoding plasmid or empty vector and the indicated NP mutants. Renilla activity was used to normalize variation in transfection efficiency. The polymerase activity in the presence of the 1918-NP was set to 100%. Error bars indicate the standard error of the mean of three independent experiments. Student's t-test was performed to determine the P value. *P<0.05, **P<0.01, ***P<0.001; NS, not significant. (PDF) [file ppat.1003279.s001.pdf]

SUPPORTING INFORMATION (Mänz et al.)

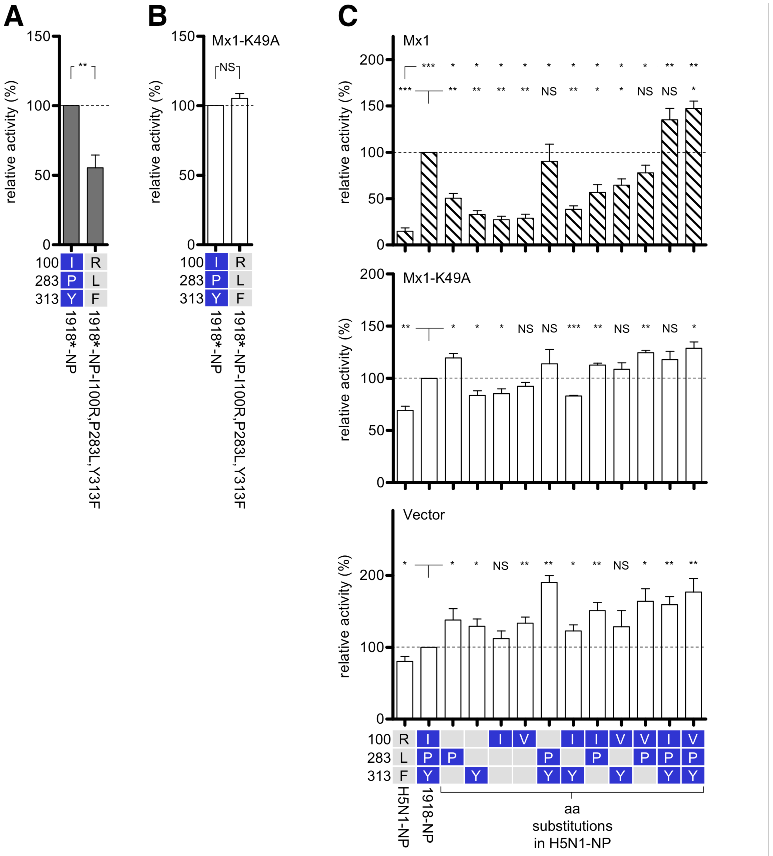

Fig. S1 Polymerase activities in the presence of Mx1 or the antivirally inactive mutant Mx1-K49A.

(A) Reporter activity of 1918 polymerase. HEK293T cells were transfected with expression plasmids coding for PB2, PB1 and PA of the pandemic 1918 strain, the indicated NP proteins, the firefly luciferase encoding minigenome, 200ng Mx1-encoding plasmid and a *Renilla*-expressing plasmid to normalize variation in transfection efficiency. Polymerase activity in the presence of antivirally inactive Mx1-K49A was used to normalize the data obtained with Mx1. Activity in the presence of the 1918*-NP was set to 100%. Error bars indicate the standard error of the mean of three independent experiments. Student's *t*-test was performed to determine the *P* value. **P<0.01.

(B) 1918 polymerase activity in the presence of the antivirally inactive mutant Mx1-K49A. HEK293T cells were transiently transfected with expression plasmids coding for the vRNP components as described in (A) including 200 ng of Mx1-K49A-encoding plasmid instead of the Mx1-coding plasmid. *Renilla* activity was used to normalize variation in transfection efficiency. The polymerase activity in the presence of the 1918*-NP was set to 100%. Error bars indicate the standard error of the mean of three independent experiments. Student's *t*-test was performed to determine the *P* value. NS, not significant.

(C) H5N1 polymerase activity in the presence of either Mx1, the antivirally inactive mutant Mx1-K49A or empty vector. HEK293T cells were transiently transfected with expression plasmids coding for the vRNP components of H5N1 including 200 ng of Mx1, Mx1-K49A-encoding plasmid or empty vector and the indicated H5N1-NP mutants. *Renilla* activity was used to normalize variation in transfection efficiency. The polymerase activity in the presence of the 1918-NP was set to 100%. Error bars indicate the standard error of the mean of three independent experiments.

Student's *t*-test was performed to determine the *P* value. *P<0.05, **P<0.01, ***P<0.001; NS, not significant.
