# Supplementary material for: Pandemic Influenza A Viruses Escape from Restriction by Human MxA through Adaptive Mutations in the Nucleoprotein
Source: PLoS Pathog. 2013 Mar 28;9(3):e1003279. doi: 10.1371/journal.ppat.1003279 (PMC3610643; doi:10.1371/journal.ppat.1003279)
Supplement: Figure S2 — Identification of amino acids in 1918 NP responsible for MxA resistance. Reporter activity of the H5N1 polymerase in HEK293T cells after co-transfection of the expression plasmids coding for MxA (200 ng) and the indicated NP proteins (100 ng). The activity in the presence of MxA was normalized to the activity observed with the antivirally inactive mutant MxA-T103A. The activity observed with the 1918*-NP was set to 100%. (PDF) [file ppat.1003279.s002.pdf]

SUPPORTING INFORMATION (Mänz et al.)

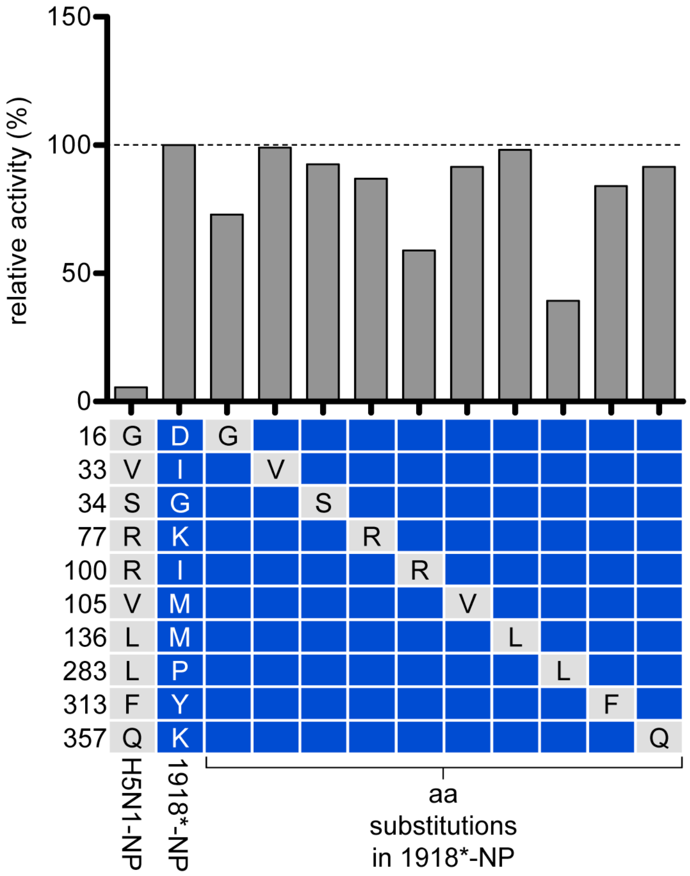

Fig. S2 Identification of amino acids in 1918 NP responsible for MxA resistance.
Reporter activity of the H5N1 polymerase in HEK293T cells after co-transfection of the expression plasmids coding for MxA (200 ng) and the indicated NP proteins (100 ng). The activity in the presence of MxA was normalized to the activity observed with the antivirally inactive mutant MxA-T103A. The activity observed with the 1918*-NP was set to 100%.
