# Supplementary material for: Pandemic Influenza A Viruses Escape from Restriction by Human MxA through Adaptive Mutations in the Nucleoprotein
Source: PLoS Pathog. 2013 Mar 28;9(3):e1003279. doi: 10.1371/journal.ppat.1003279 (PMC3610643; doi:10.1371/journal.ppat.1003279)
Supplement: Figure S3 — Polymerase activities in the presence of the antivirally inactive mutant MxA-T103A. H5N1 polymerase reporter activity was determined after co-transfection of expression plasmids coding for the indicated NP mutants (100 ng) and the antivirally inactive mutant MxA-T103A (200 ng). The reporter activity observed with the 1918-NP was set to 100%. Error bars indicate the standard error of the mean of three independent experiments. Student's t-test was performed to determine the P value. *P<0.05, **P<0.01; NS, not significant. (PDF) [file ppat.1003279.s003.pdf]

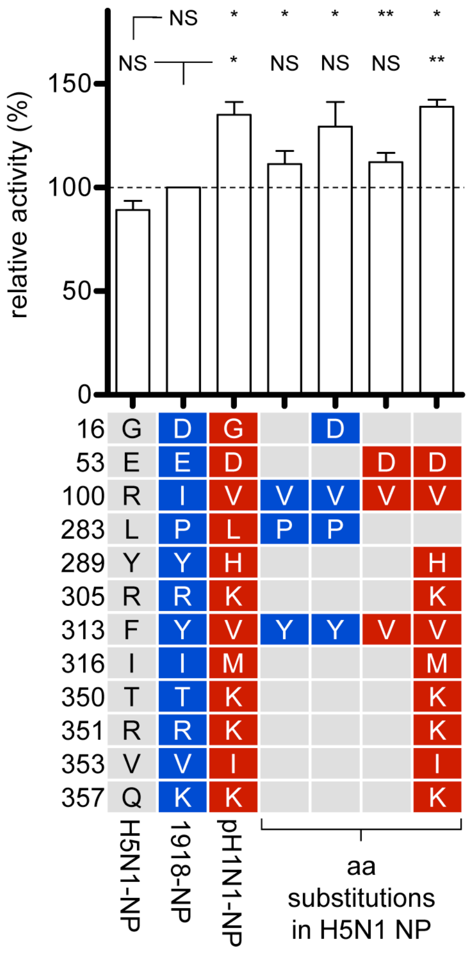

Fig. S3 Polymerase activities in the presence of the antivirally inactive mutant MxA-T103A.

H5N1 polymerase reporter activity was determined after co-transfection of expression plasmids coding for the indicated NP mutants (100 ng) and the antivirally inactive mutant MxA-T103A (200 ng). The reporter activity observed with the 1918-NP was set to 100%. Error bars indicate the standard error of the mean of three independent experiments. Student's *t*-test was performed to determine the *P* value. *P<0.05, **P<0.01; NS, not significant.
