# Supplementary material for: Pandemic Influenza A Viruses Escape from Restriction by Human MxA through Adaptive Mutations in the Nucleoprotein
Source: PLoS Pathog. 2013 Mar 28;9(3):e1003279. doi: 10.1371/journal.ppat.1003279 (PMC3610643; doi:10.1371/journal.ppat.1003279)
Supplement: Figure S4 — Localization of the adaptive mutation 16D in NP. The model for the full-length structure of H5N1 NP (A/Thailand/1(KAN-1)/04) harboring the mutations G16D, R100I, L283P and F313Y was generated utilizing I-TASSER. The N-terminus that was not resolved in the crystal structure (aa 1–20) is highlighted in light orange, whereas 1918-specific amino acids that confer Mx resistance are shown in blue (16D, 100I/V, 283P and 313Y). (PDF) [file ppat.1003279.s004.pdf]

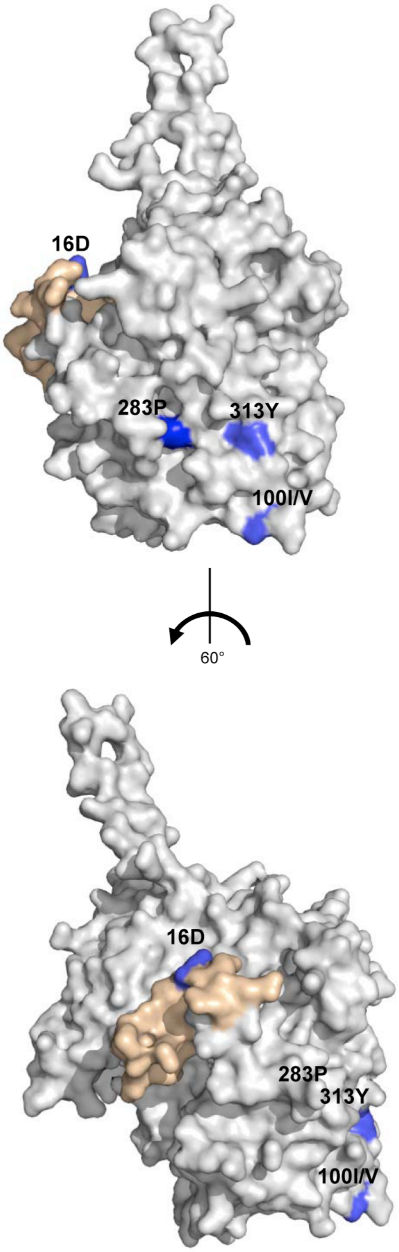

Fig. S4 Localization of the adaptive mutation 16D in NP.
The model for the full-length structure of H5N1 NP (A/Thailand/1(KAN-1)/04) harboring the mutations G16D, R100I, L283P and F313Y was generated utilizing I-TASSER. The N-terminus that was not resolved in the crystal structure (aa 1-20) is highlighted in light orange, whereas 1918-specific amino acids that confer Mx resistance are shown in blue (16D, 100I/V, 283P and 313Y).
