# Supplementary material for: Pandemic Influenza A Viruses Escape from Restriction by Human MxA through Adaptive Mutations in the Nucleoprotein
Source: PLoS Pathog. 2013 Mar 28;9(3):e1003279. doi: 10.1371/journal.ppat.1003279 (PMC3610643; doi:10.1371/journal.ppat.1003279)
Supplement: Figure S5 — Phylogenetic analysis of representative NP sequences and the presence or loss of Mx-resistance enhancing mutations. The maximum likelihood tree of 147 aligned sequences shows four genotypes, i.e., (i) the human H1N1, H2N2 and H3N2 viruses, (ii) the classical swine H1N1 viruses and pandemic (2009) H1N1 viruses, (iii) the European lineages of swine influenza viruses, and (iv) the North American avian influenza viruses. Strain designations and GenBank acc. nos. are presented. Numbers at nodes indicate bootstrap values obtained after 1,000 replications. Only bootstrap values greater than 50% are presented. The bar indicates substitutions per site. Alterations of amino acid positions shown to influence Mx resistance (Figure 3, Figure S9) are highlighted in bold. (PDF) [file ppat.1003279.s005.pdf]

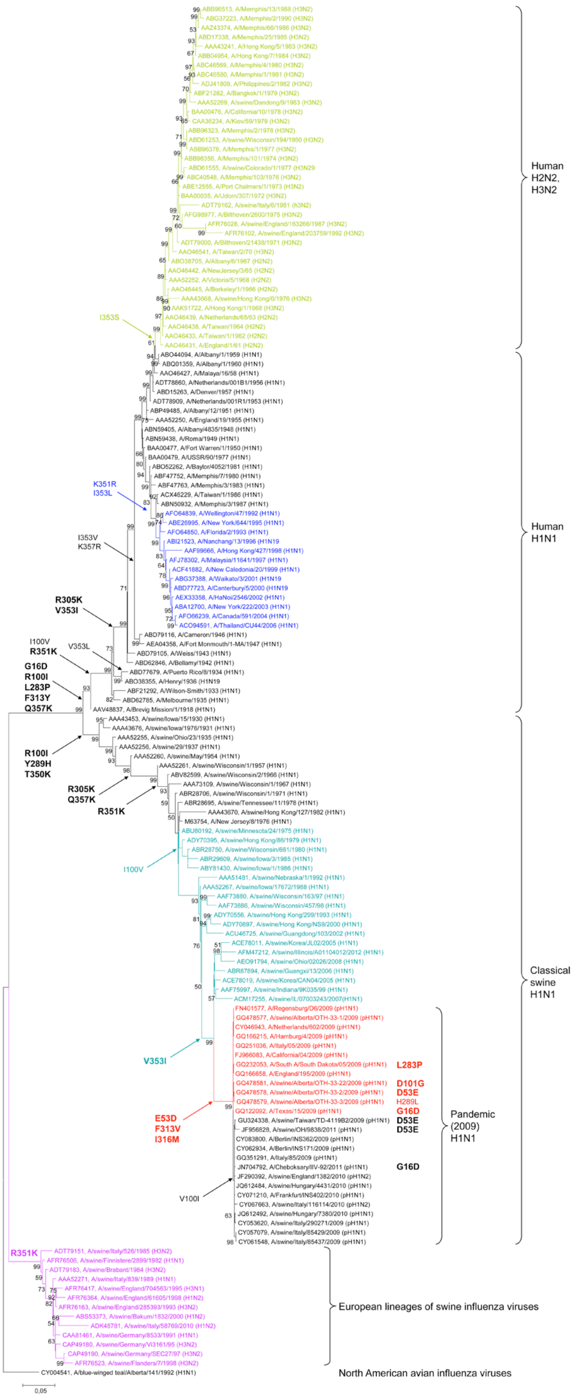

Fig. S5 Phylogenetic analysis of representative NP sequences and the presence or loss of Mx-resistance enhancing mutations.
The maximum likelihood tree of 147 aligned sequences shows four genotypes, i.e., (i) the human H1N1, H2N2 and H3N2 viruses, (ii) the classical swine H1N1 viruses and pandemic (2009) H1N1 viruses, (iii) the European lineages of swine influenza viruses, and (iv) the North American avian influenza viruses. Strain designations and GenBank acc. nos. are presented. Numbers at nodes indicate bootstrap values obtained after 1,000 replications. Only bootstrap values greater than 50% are presented. The bar indicates substitutions per site. Alterations of amino acid positions shown to influence Mx resistance (Fig. 3, Fig. S9) are highlighted in bold.
