# Supplementary material for: Pandemic Influenza A Viruses Escape from Restriction by Human MxA through Adaptive Mutations in the Nucleoprotein
Source: PLoS Pathog. 2013 Mar 28;9(3):e1003279. doi: 10.1371/journal.ppat.1003279 (PMC3610643; doi:10.1371/journal.ppat.1003279)
Supplement: Figure S6 — Mx resistance of NP variants of swine origin. (A–B) H5N1 or (C) pH1N1 polymerase activity in the presence of human MxA (A and C) or porcine Mx1 (poMx1) (B). The activity in the presence of human MxA or poMx1 was normalized to the activity observed after co-expression of the antivirally inactive MxA mutant MxA-T103A. The activity observed with the pH1N1-NP was set to 100%, respectively. Error bars indicate the standard error of the mean of three independent experiments. Student's t-test was performed to determine the P value. **P<0.01, ***P<0.001; NS, not significant. (PDF) [file ppat.1003279.s006.pdf]

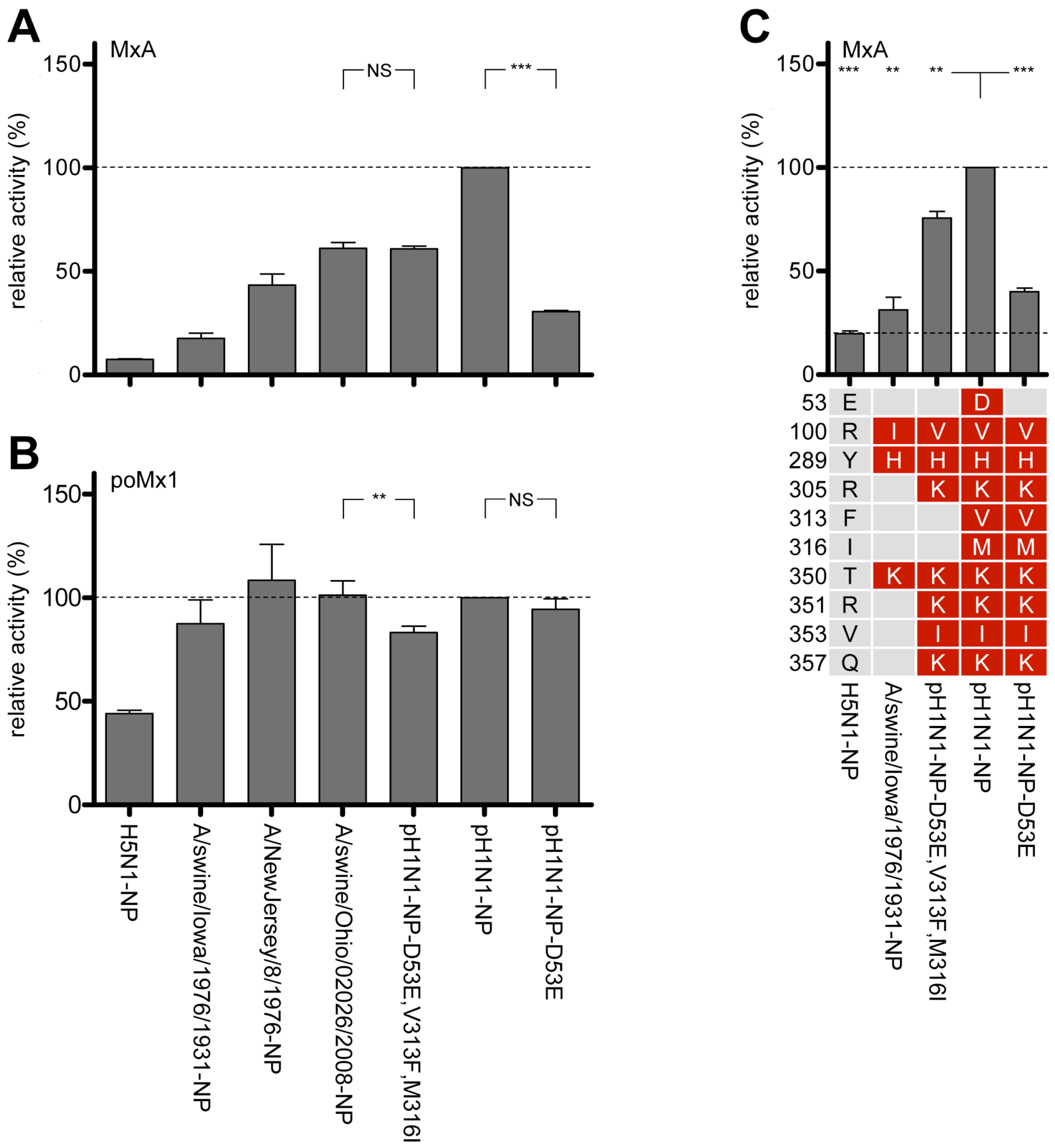

Fig. S6 Mx resistance of NP variants of swine origin.
(A-B) H5N1 or **(C)** pH1N1 polymerase activity in the presence of human MxA (A and C) or porcine Mx1 (poMx1) (B). The activity in the presence of human MxA or poMx1 was normalized to the activity observed after co-expression of the antivirally inactive MxA mutant MxA-T103A. The activity observed with the pH1N1-NP was set to 100%, respectively. Error bars indicate the standard error of the mean of three independent experiments. Student's *t*-test was performed to determine the *P* value. ***P*<0.01, ****P*<0.001; NS, not significant.
