# Supplementary material for: Pandemic Influenza A Viruses Escape from Restriction by Human MxA through Adaptive Mutations in the Nucleoprotein
Source: PLoS Pathog. 2013 Mar 28;9(3):e1003279. doi: 10.1371/journal.ppat.1003279 (PMC3610643; doi:10.1371/journal.ppat.1003279)
Supplement: Figure S7 — Inhibition of polymerase activities by porcine and canine Mx proteins. (A) Swine NPTr or NSK cells were transfected with expression plasmids coding for NP, PB2, PB1 and PA of H5N1, the porcine Pol1-driven firefly luciferase encoding minigenome, 200 ng of either porcine Mx1 (poMx1), MxA, or the GTPase-inactive MxA mutant MxA-T103A and a Renilla-expressing plasmid to normalize variation in transfection efficiency. The activity in the presence of MxA-T103A was set to 100%. Error bars indicate the standard error of the mean of three independent experiments. Student's t-test was performed to determine the P value. **P<0.01; ***P<0.001. (B) HEK293T cells were transfected with expression plasmids coding for NP, PB2, PB1 and PA of H5N1, the firefly luciferase encoding minigenome, 200 ng of either canine Mx1 (cMx1), canine Mx2 (cMx2), MxA or the GTPase-inactive MxA mutant MxA-T103A and a Renilla-expressing plasmid to normalize variation in transfection efficiency. The activity in the presence of MxA-T103A was set to 100%. Error bars indicate the standard error of the mean of three independent experiments. Student's t-test was performed to determine the P value. ***P<0.001; NS, not significant. (PDF) [file ppat.1003279.s007.pdf]

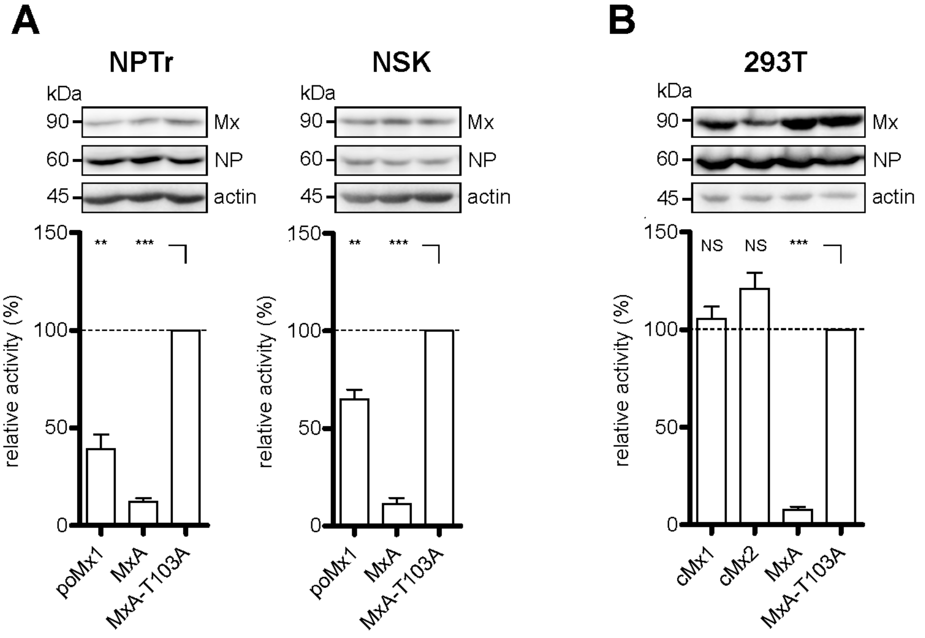

Fig. S7 Polymerase activities of porcine and canine Mx proteins

(A) Swine NPTr or NSK cells were transfected with expression plasmids coding for NP, PB2, PB1 and PA of H5N1, the porcine Pol1-driven firefly luciferase encoding minigenome, 200ng of either porcine Mx1 (poMx1), MxA, or the GTPase-inactive MxA mutant MxA-T103A and a *Renilla*-expressing plasmid to normalize variation in transfection efficiency. The activity in the presence of MxA-T103A was set to 100%. Error bars indicate the standard error of the mean of three independent experiments. Student's *t*-test was performed to determine the *P* value. $^{**}P<0.01$; $^{***}P<0.001$.

(B) HEK293T cells were transfected with expression plasmids coding for NP, PB2, PB1 and PA of H5N1, the firefly luciferase encoding minigenome, 200ng of either canine Mx1 (cMx1), canine Mx2 (cMx2), MxA or the GTPase-inactive MxA mutant MxA-T103A and a Renilla-expressing plasmid to normalize variation in transfection efficiency. The activity in the presence of MxA-T103A was set to 100%. Error bars indicate the standard error of the mean of three independent experiments. Student's *t*-test was performed to determine the *P* value. $^{***}P<0.001$; NS, not significant.
