# Supplementary material for: Pandemic Influenza A Viruses Escape from Restriction by Human MxA through Adaptive Mutations in the Nucleoprotein
Source: PLoS Pathog. 2013 Mar 28;9(3):e1003279. doi: 10.1371/journal.ppat.1003279 (PMC3610643; doi:10.1371/journal.ppat.1003279)
Supplement: Figure S8 — Mx resistance-enhancing mutations influence transcription and viral growth in avian cells. (A) Comparison of viral transcription in MDCKII or avian LMH cells infected with an MOI of 5 of either H5N1 (KAN-1) or H5N1-NP-R100I,F313Y after the indicated hours post infection (h.p.i.). mRNA, cRNA, and vRNA levels were determined using primer extension analysis with primers specific for segment 6. Levels of cellular 5sRNA served as internal control. (B) Avian LMH cells were infected with an MOI of 0.001 of either H5N1 or H5N1-NP-R100I,F313Y and incubated at 37°C. At the indicated time points post infection (p.i.), virus titers were determined by plaque assay. Error bars indicate the standard error of the mean of three independent experiments. (PDF) [file ppat.1003279.s008.pdf]

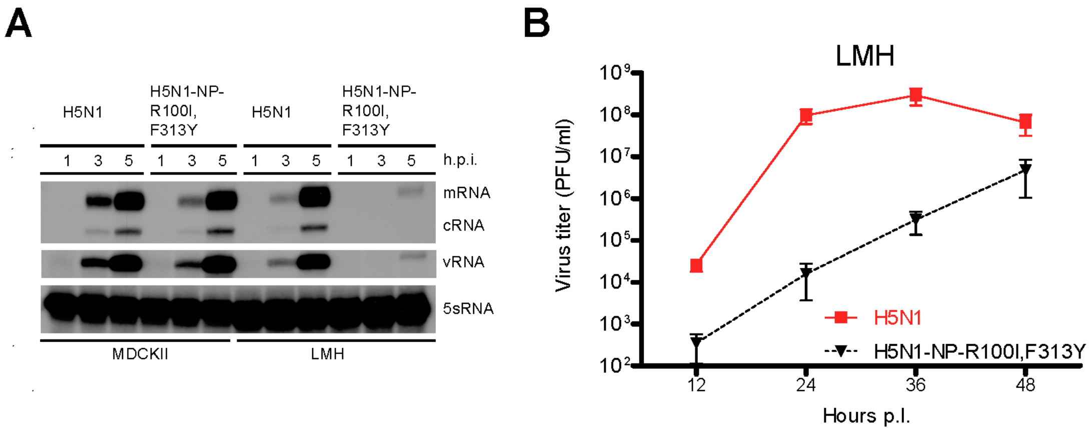

Fig. S8 Mx resistance-enhancing mutations influence transcription and viral growth in avian cells

(A) Comparison of viral transcription in MDCKII or avian LMH cells infected with an MOI of 5 of either H5N1 (KAN-1) or H5N1-NP-R100I,F313Y after the indicated hours post infection (h.p.i.). mRNA, cRNA, and vRNA levels were determined using primer extension analysis with primers specific for segment 6. Levels of cellular 5sRNA served as internal loading control.

(B) Avian LMH cells were infected with an MOI of 0.001 of wild-type or the indicated H5N1 mutant viruses and incubated at 37°C. At the indicated time points post infection (p.i.), virus titers were determined by plaque assay. Error bars indicate the standard error of the mean of three independent experiments.
