# Supplementary material for: Pandemic Influenza A Viruses Escape from Restriction by Human MxA through Adaptive Mutations in the Nucleoprotein
Source: PLoS Pathog. 2013 Mar 28;9(3):e1003279. doi: 10.1371/journal.ppat.1003279 (PMC3610643; doi:10.1371/journal.ppat.1003279)
Supplement: Figure S9 — Mutation D101G in NP of pH1N1 strongly reduces resistance to MxA. H5N1 polymerase activity after co-transfection of MxA (200 ng) and the indicated NP (100 ng) expression plasmids. The activity in the presence of MxA was normalized to the activity observed with the antivirally inactive mutant MxA-T103A. The reporter activity observed with the pH1N1-NP in the presence of MxA was set to 100%. Error bars indicate the standard error of the mean of two-three independent experiments. (PDF) [file ppat.1003279.s009.pdf]

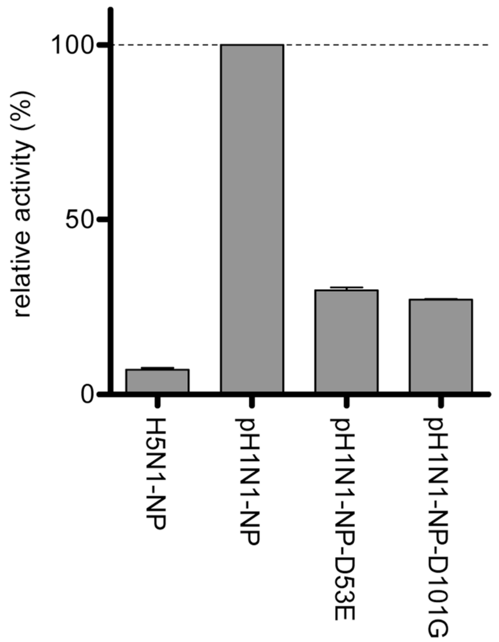


**Fig. S9 Mutation D101G in NP of pH1N1 strongly reduces resistance to MxA.**
H5N1 reporter activity after co-transfection of MxA (200 ng) and the indicated NP (100 ng) expression plasmids. The activity in the presence of MxA was normalized to the activity observed with the antivirally inactive mutant MxA-T103A. The reporter activity observed with the pH1N1-NP in the presence of MxA was set to 100%. Error bars indicate the standard error of the mean of two-three independent experiments.
